# JNK Controls the Onset of Mitosis in Planarian Stem Cells and Triggers Apoptotic Cell Death Required for Regeneration and Remodeling

**DOI:** 10.1371/journal.pgen.1004400

**Published:** 2014-06-12

**Authors:** María Almuedo-Castillo, Xenia Crespo, Florian Seebeck, Kerstin Bartscherer, Emili Salò, Teresa Adell

**Affiliations:** 1 Department of Genetics and Institute of Biomedicine, University of Barcelona, Barcelona, Catalonia, Spain; 2 Max Planck Research Group Stem Cells and Regeneration, Max Planck Institute for Molecular Biomedicine, Münster, Germany; 3 Faculty of Medicine, University of Münster, Münster, Germany; University of Oxford, United Kingdom

## Abstract

Regeneration of lost tissues depends on the precise interpretation of molecular signals that control and coordinate the onset of proliferation, cellular differentiation and cell death. However, the nature of those molecular signals and the mechanisms that integrate the cellular responses remain largely unknown. The planarian flatworm is a unique model in which regeneration and tissue renewal can be comprehensively studied *in vivo*. The presence of a population of adult pluripotent stem cells combined with the ability to decode signaling after wounding enable planarians to regenerate a complete, correctly proportioned animal within a few days after any kind of amputation, and to adapt their size to nutritional changes without compromising functionality. Here, we demonstrate that the stress-activated c-jun–NH_2_–kinase (JNK) links wound-induced apoptosis to the stem cell response during planarian regeneration. We show that JNK modulates the expression of wound-related genes, triggers apoptosis and attenuates the onset of mitosis in stem cells specifically after tissue loss. Furthermore, in pre-existing body regions, JNK activity is required to establish a positive balance between cell death and stem cell proliferation to enable tissue renewal, remodeling and the maintenance of proportionality. During homeostatic degrowth, *JNK* RNAi blocks apoptosis, resulting in impaired organ remodeling and rescaling. Our findings indicate that JNK-dependent apoptotic cell death is crucial to coordinate tissue renewal and remodeling required to regenerate and to maintain a correctly proportioned animal. Hence, JNK might act as a hub, translating wound signals into apoptotic cell death, controlled stem cell proliferation and differentiation, all of which are required to coordinate regeneration and tissue renewal.

## Author Summary

Planarians, thanks to their extraordinary regenerative capacity, represent a unique model of animal regeneration. After amputation, new animals regenerate from each individual piece of tissue, leading Dalyell to describe them as “immortal under the edge of the knife” in 1814. Planarians also continuously renew their tissues and adapt their size in accordance with nutritional supply. This amazing plasticity relies on the presence of a population of adult pluripotent stem cell, the neoblasts. However, little is known about the mechanisms that trigger cell responses, such as cell death and division, which are required to regenerate and maintain tissues and organs in response to injury or nutritional challenge. Here, we show that JNK acts as a hub in the coordination of these events. Specifically in response to tissue loss, JNK modulates the expression of wound-related genes, induces the elimination of unnecessary cells by apoptotic cell death and controls cell division in neoblasts. Loss of JNK function results in the deregulation of these processes and prevents regeneration. Moreover, we demonstrate that JNK-dependent apoptosis is crucial to generate proportioned organisms during tissue turnover. Our findings reveal a central mechanism in planarians that senses tissue loss and translates this information into cellular responses leading to regeneration and tissue renewal.

## Introduction

The regeneration of missing tissues requires tight coordination between stem cell proliferation, differentiation, and cell death. However, it remains unclear how these processes are integrated to generate a well-proportioned organism. We addressed this question using the freshwater planarian *Schmidtea mediterranea*, a popular model system in regeneration research. These animals stand out in housing a pluripotent cell population (neoblasts) throughout their lives [1]–[7]. Due to their pluripotent nature, neoblasts confer planarians with unmatched plasticity, allowing them to regenerate any body part within a few days and to continuously modulate their size in accordance with energy supply while sustaining physiological functions [8]–[12]. Planarians thus are a unique model in which to study the molecular processes that underlie regeneration *in viv*o. After any kind of amputation in planarians, the wound is closed by muscle contraction within a few minutes [13], [14]. Subsequent signaling from the wound area triggers specific gene activation [15], [16], the induction of apoptotic cell death [17] and the controlled induction of neoblast proliferation and differentiation [18], [19].

JNK is a stress-activated protein kinase (SAPK) that belongs to a large family of mitogen-activated protein kinases (MAPKs) and regulates essential cellular processes, such as stem cell proliferation, differentiation and programmed cell death, in response to stress [20], [21]. As a stress indicator, JNK has been implicated in cell cycle regulation, where it ensures the controlled onset of mitosis [22], [23]. Deregulation of JNK-mediated signaling has been demonstrated in a wide variety of human diseases, including neurodegenerative disorders, diabetes and cancer [20], [24]. Transcriptional profile analyses have identified planarian orthologs of downstream effectors of the JNK pathway such as *jun* and *fos* as possible participants in neoblast maintenance [7] and in the wound response program [16].

Here we show that loss of function of the *S. mediterranea JNK* ortholog after RNA interference (RNAi) prevents the regeneration of missing structures. In response to wounding, *JNK(RNAi)* planarians exhibited decreased expression of wound-induced genes, a severe attenuation of the apoptotic response and acceleration of the dynamics of neoblast proliferation between G2- to M-phase transition. In pre-existing regions, the positive balance between cell death and stem cell proliferation was reversed, leading to improper remodeling and rescaling in *JNK(RNAi)* animals. Furthermore, *JNK* RNAi specifically interfered with the maintenance of body proportion during degrowth, but not growth, as only decreases in size were dependent on the activation of apoptosis. These findings point to JNK as an essential stress response element required for the integration and coordination of the apoptotic and proliferative responses triggered by tissue loss to ensure successful regeneration and tissue remodeling. Moreover, our results contribute to a novel aspect of regeneration: the importance of temporal control of the cell cycle progression of stem cells for balanced differentiation [25].

## Results

### JNK is required for proper regeneration of missing structures

**Figure 1:**
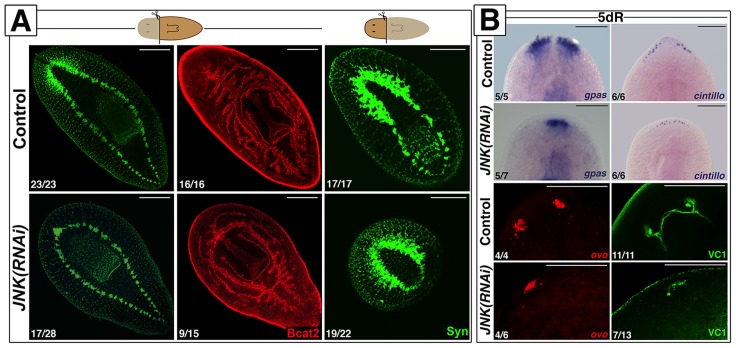
JNK is required for anterior and posterior regeneration. (**A**) Immunostaining with anti-synapsin (Syn) and anti-β-catenin-2 (Bcat2), which allow the visualization of the central nervous and digestive systems, respectively, in regenerating trunk and head fragments 15 days after anterior amputation. (Top left, anterior). (**B**) Expression analysis by WISH of the brain branches (*gpas*+), the anterior chemoreceptors (*cintillo*+) and FISH analysis of the expression of eye/eye progenitor cells (*ovo*+). The visual system was visualized by immunostaining with anti-VC1(VC-1). (Top, anterior). The number of representative samples with respect to the total is indicated in each image Syn, BCat2 and VC1 images correspond to confocal z-projections. Scale bars: 300 µm (A), 200 µm (B). dR, days of regeneration.

We identified a single JNK ortholog in the *S. mediterranea* genome (*Smed-JNK*) (Ref for genome, or weblink to Washington) (see alignment in Figure S1A). We performed RNAi of *JNK* to decipher its function during planarian regeneration (Figure S1B-S1C). After head amputation, trunk fragments were unable to regenerate anterior structures such as the brain and the anterior digestive branch. Similarly, head fragments failed to regenerate medial and posterior structures, including pharynx and tail (Figure 1A). Analysis of the pattern of several differentiated structures, such as brain branches (*gpas*+) [26], anterior chemoreceptors (*cintillo*+) [27] and the visual system (*ovo*+ and VC1+) [28], [29], revealed aberrant regeneration in the anterior blastema of *JNK(RNAi)* planarians (Figure 1B). We next investigated whether this inability to regenerate was associated with a prior defect in polarity determination. In control animals, a few hours after injury, the expression of the polarity genes (*notum*, *sFRP1*, *wnt1*) is activated in the wound region, and is subsequently polarized and confined to the posterior or anterior midline regions [30]–[32]. The initial induction of these polarity genes is irradiation insensitive and thus stem cell independent, whereas the polarization of their expression domains relies on stem cell proliferation [31], [32]. Whole-mount *in situ* hybridization (WISH) analysis revealed that the early expression of *notum* (18 hours) and *sFRP1* (24 hours) in anterior wounds of *JNK(RNAi)* animals was indistinguishable from that of control animals (Figure S2) [30]–[32], as was the early expression (24 hours) of *wnt1* in both anterior and posterior wounds [31] (Figure S2). However, the subsequent polarized and confined expression of these genes was severely attenuated in *JNK(RNAi)* animals (Figure S2). Fading of the anterior expression of *wnt1*, which occurs around 48 h after injury and is known to be stem cell independent [32], was also observed in *JNK(RNAi)* animals (Figure S2). Thus, while the initial establishment of polarity by differentiated cells is JNK-independent, JNK is specifically required for the maintenance of polarity gene expression during later, stem cell-dependent stages.

Together, these results demonstrate that JNK is required for proper regeneration of missing tissues independently of the initial specification of identity.

### JNK modulates early gene expression triggered after wound closure

**Figure 2:**
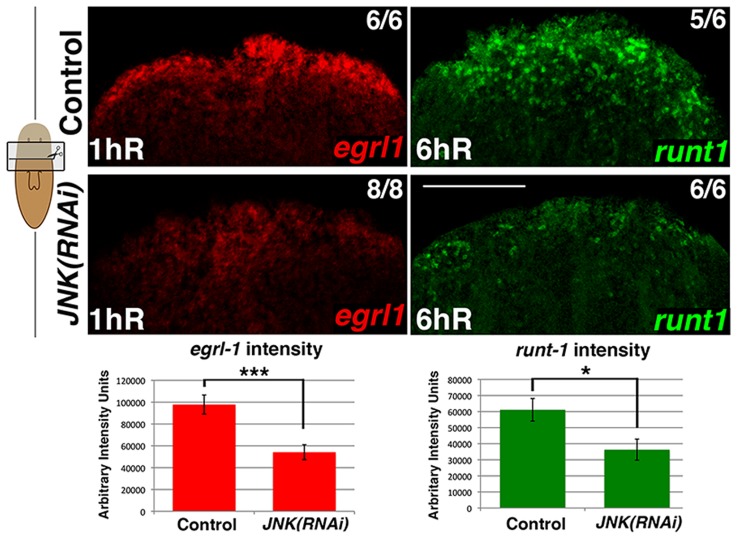
JNK modulates early wound-induced gene expression. FISH analysis of *egrl1* and *runt1* expression in trunk fragments in response to wounding (after anterior amputation) and quantification of *egrl1* and *runt1* expression. At least five biological replicates were used. (Top, anterior). Error bars represent the standard error of the mean. Scale bars: 200 µm. hR, hours of regeneration.

Early wound-generated signaling has been proposed to mediate the onset of regeneration after injury [15], [16], however the underlying molecular mechanisms remain largely unknown. To assess a putative role of JNK signaling in wound healing, we analyzed the dynamics of wound closure and quantified the expression of the early wound-induced genes *egrl1* and *runt1* [15], [16] after *JNK* RNAi. Although normal wound closure was observed in *JNK(RNAi)* animals, the blastema shape and size were aberrant (Figure S3), and expression of these early wound-induced genes was diminished (Figure 2-S4), indicating that JNK might be important for proper signal transmission or interpretation at the wound.

### JNK attenuates the cell cycle progression of planarian stem cells between G2- to M-phase transition

**Figure 3:**
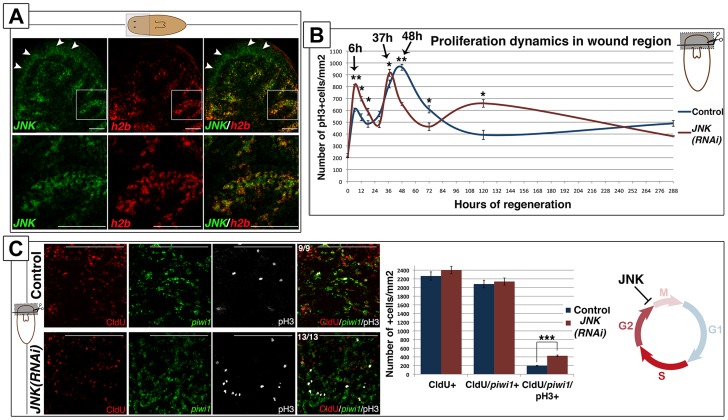
JNK attenuates the cell cycle progression of planarian neoblasts. (**A**) Double FISH analysis showing *JNK* and *h2b* expression in anterior region of intact animals. White arrowheads point to the sole expression of *JNK* in the brain. White boxes point to the region corresponding to the magnifications showed bellow where *JNK* are *h2b* are coexpressed in neoblast cells between the gut branches. (**B**) Graph depicting the quantity of mitotic cells (pH3+) in the wound region during anterior regeneration. At least nine biological replicates were used per time point. (**C**) Triple labeling of CldU+, *piwi1*+ and pH3+ cells in the wound region 6 hours after anterior amputation. To note all the pH3+(white) cells were also CldU+(red). Quantifications of the number of these single CldU+, double CldU+/*piwi1*+ and triple CldU+/*piwi1*+/pH3+ cells are shown. At least nine biological replicates were used. All images correspond to confocal z-projections. (Top left, anterior). Error bars represent the standard error of the mean. Data were analyzed by Student's t-test. *P<0.05; **P<0.01; ***P<0.001; differences are considered significant at P<0.05. Scale bars: 100 µm (B), 50 µm (C). hR, hours of regeneration; dR, days of regeneration.

Neoblast are the only mitotically active somatic cells in the adult planarian [33]. Double fluorescent whole-mount *in situ* hybridization (FISH) analysis of *JNK* and the neoblast marker *histone 2B* (*h2b*) [33] showed that *JNK* is expressed in *h2b*-negative differentiated tissues, such as the brain, and in *h2b*-positive neoblasts (Figure 3A). Accordingly, *JNK* expression vanishes specifically in the subpopulation of proliferative cells after eliminating neoblasts by h2b RNAi [33] (Figure S5A) or by irradiation [34] (Figure S5B). Expression analysis of *h2b* by FISH and qRT-PCR showed that the proportion of neoblasts in *JNK(RNAi)* animals was similar to that of controls (Figure S5C-S5D). In agreement, sorting of the different planarian cell populations by FACS, a method by which planarian cells can be separated based on their DNA content and size [35], [36], showed no alterations in the proportion of actively cycling cells (in the S or G2/M phase of the cell cycle; ×1 sub-population) after *JNK* RNAi (Figure S5E). Therefore, although JNK is expressed in neoblasts and essential for regeneration, it is not required for their viability.

Amputation in planarians triggers two waves of neoblast proliferation in a temporally coordinated manner [18], [19], [37], [38]. The first proliferative peak can be detected 6 hours after amputation in response to any type of injury, whereas the second peak, which appears 48 hours after amputation, is only observed after injuries that involve tissue loss and subsequent neoblast recruitment to the wound area [19]. We performed a thorough analysis of the mitotic response in the wound region after anterior amputation by quantifying pH3-positive neoblasts. Although *JNK(RNAi)* animals displayed a bimodal mitotic response after wounding, the temporal dynamic of the response was altered (Figure 3B-S5F). During the first mitotic response (6 hours after amputation), we observed a significant increase in the number of neoblasts entering mitosis as compared with controls (Figure 3B). Furthermore, the second mitotic peak in JNK(RNAi) animals was sharper and occurred 37 hours after amputation, 10 hours earlier than in control animals, indicating an earlier onset of mitosis. Finally, we observed an increase in the number of mitotic neoblasts 5 days after amputation in *JNK(RNAi)* animals (Figure 3B). A similar alteration was observed in the wound region of *JNK(RNAi)* animals after posterior amputation (Figure S5G). Quantification of the number of mitotic cells using an alternative method, a modified Gomori technique [18], revealed an alteration in the mitotic profile of *JNK(RNAi)* animals comparable to that demonstrated by quantifying the number of pH3+ cells (Figure S5H).

To further characterize the alteration of the cell cycle dynamics after *JNK* RNAi, we labeled cells in the S-phase of the cell cycle by injecting planarians with the thymidine analogue chlorodeoxyuridine (CldU) before amputation. Detection of CldU labeling in cells expressing the neoblast marker *piwi-1* [34] allowed us to specifically quantify the number of neoblasts that went through S-phase but did not start to differentiate at the time of fixation (16h post-CldU injection and 6h post-amputation). Quantification of CldU/*piwi1*+ cells, as well as of CldU+ cells, revealed no differences between the control and *JNK(RNAi)* animals (Figure 3C), indicating that JNK is not required for normal progression through S phase. Conversely, quantification of CldU/*piwi1*+ cells positive for the mitotic marker pH3 (CldU+/*piwi1*+/pH3+) corroborated the significant increase in mitotic cells in *JNK(RNAi)* animals (Figure 3C). The maintenance of the total CldU+ cells shows that the increase of mitotic cells in *JNK(RNAi)* is not explained by a previous increase in S-phase cells, but by a shorter G2 phase and hence a faster entry into mitosis.

FISH and qRT-pCR analysis of post-mitotic neoblast progeny markers - referred to as early (*NB.32.1g*) and late (*Agat-1*) division progeny genes [39] - revealed that the number of cells expressing these markers and their expression levels were unaltered between control and *JNK(RNAi)* animals in any of the regions analyzed (Figures S6). This suggests that the acceleration of the cell cycle of *JNK(RNAi)* neoblasts does not alter their capacity to exit the proliferative state and produce progeny.

Altogether, these results indicate that, although *JNK* is expressed in neoblasts, it is not essential for their viability but for the control of their cell cycle length. Hence, in planarians, JNK controls the wound-induced proliferative response attenuating the transition between G2- to M-phase.

### JNK is required for apoptotic cell death and to restore body proportion after amputation

**Figure 4:**
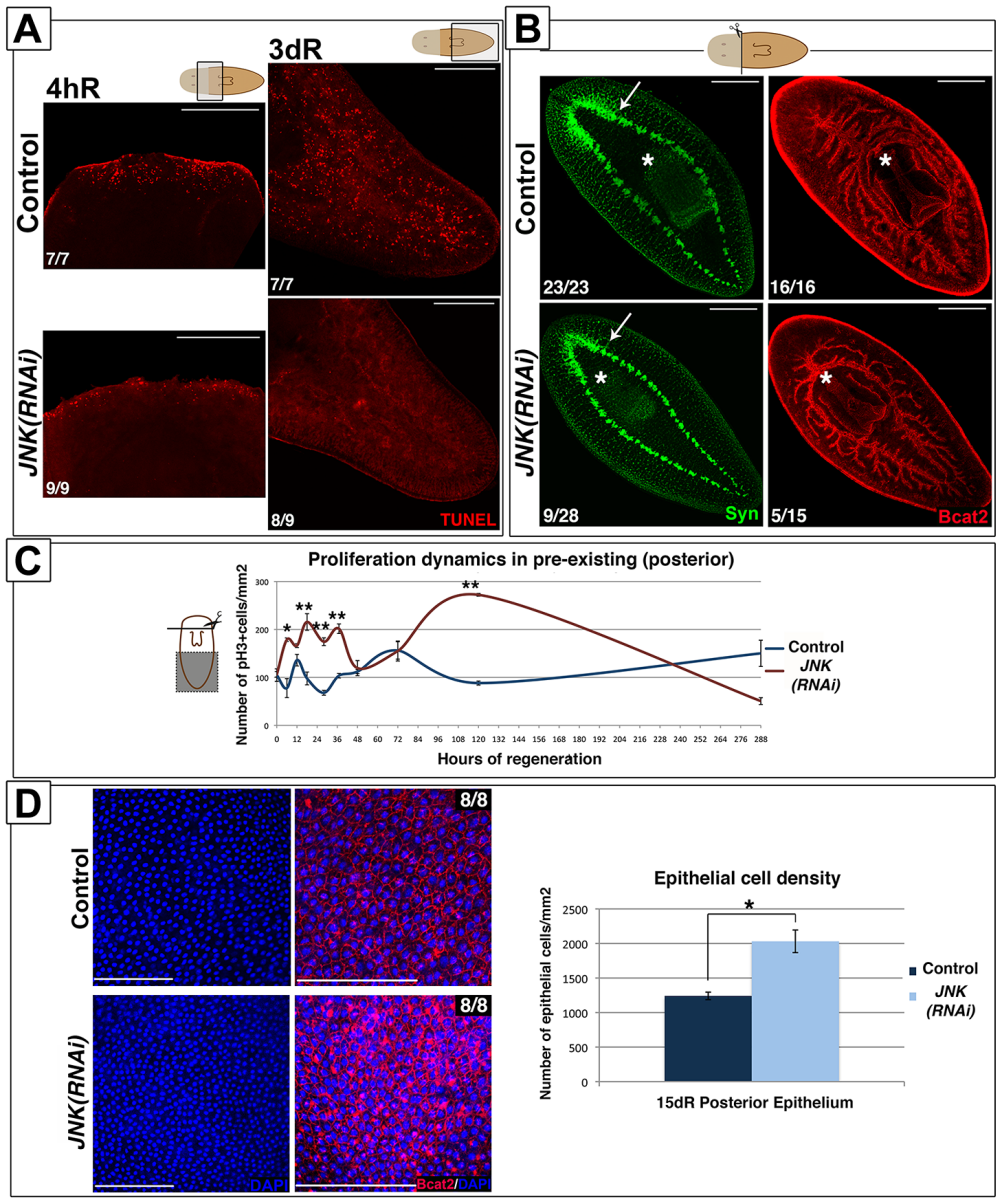
JNK triggers apoptosis and restores proportionality after amputation. (**A**) Whole-mount TUNEL staining showing apoptotic cells in the wound region (4 hR) and in pre-existing regions (3 dR) after anterior amputation. (Top/top left, anterior). (**B**) Immunostaining with anti-synapsin (Syn) and anti-β-catenin-2 (Bcat2) antibodies in regenerating trunk fragments 15 days after anterior amputation. These animals are the same as those used in the experiment depicted in Figure 1A. The white asterisk and arrow indicate the pharynx and the distal-most part of the brain, respectively. (Top left, anterior). (**C**) Graph showing the quantity of mitotic cells (pH3+) in pre-existing (posterior) regions after anterior regeneration. At least nine biological replicates were used per time point. (**D**) DAPI and BCat-2 staining of the epithelia (Top left, anterior) and quantification of the number of epithelial cells/mm^2^ in these post-pharyngeal (pre-existing) regions in regenerating trunks 15 days after amputation. Eight biological replicates were used per time point. All images correspond to confocal z-projections. Error bars represent standard error of the mean. Data were analyzed by Student's t-test. *P<0.05; **P<0.01; differences are considered significant at P<0.05. Scale bars: 200 µm (A), 300 µm (B), 50 µm (D). hR, hours of regeneration; dR, days of regeneration.

Cell death is necessary for tissue remodeling during planarian regeneration [17], [40]. In planarians, amputation triggers two peaks of apoptotic cell death, one 4 hours after injury, which is localized in the wound region, and a second peak 3 days after amputation that spreads throughout the organism [17]. These apoptotic responses are stem cell-independent and thus, occur almost exclusively in post-mitotic cells [17]. Using the TUNEL assay, we analyzed apoptotic cell death after anterior amputation in whole-mount preparations and tissue sections, and found that *JNK* RNAi prevents the activation of apoptosis both in regenerating and pre-existing regions (Figure 4A-S7A-S7B). Analysis of mitotic rates during the regenerative process in pre-existing regions revealed an altered profile with a general increment in the number of mitotic cells in *JNK(RNAi)* animals with respect to control counterparts (Figure 4C), an effect that was also observed after posterior amputation (Figure S7C). Thus, in pre-existing regions in which remodeling is required, *JNK(RNAi)* planarians exhibited a complete inhibition of the apoptotic response, together with an increase in the rate of proliferation. This inversion of the balance between cell death and cell proliferation in *JNK(RNAi)* animals prevents the restoration of correct body proportion, as evidenced by their inability to re-adjust the position of pre-existing organs such as the pharynx as regeneration proceeds (Figure 4B). The disruption of rescaling is not due to a complete inability to regenerate, as these defects were also observed in the less penetrant *JNK(RNAi)* phenotypes, which can at least partially regenerate anterior structures such as the brain (Figure 4B).

The lack of apoptosis in pre-existing regions should generate a larger number of cells than found in controls. To determine the fate of these cells we quantified the number of DAPI-stained epidermal cells. Interestingly, the number of epidermal cells was increased in the posterior epithelium of *JNK(RNAi)* versus control animals (Figure 4D), suggesting that an increased number of cells might contribute to the epidermis, and possible other tissues.

These findings indicate that JNK activity is essential to trigger wound-induced apoptotic cell death as well as the second systemic apoptotic response during planarian regeneration. Inhibition of JNK-dependent apoptosis and probably together with the increase in proliferative rates, prevents remodeling of pre-existing regions and causes the higher density of epithelial cells. Importantly, these results demonstrate that in planarians, the induction of neoblast proliferation might be independent of apoptotic cell death.

### JNK requirements are specific to injuries involving tissue loss

**Figure 5:**
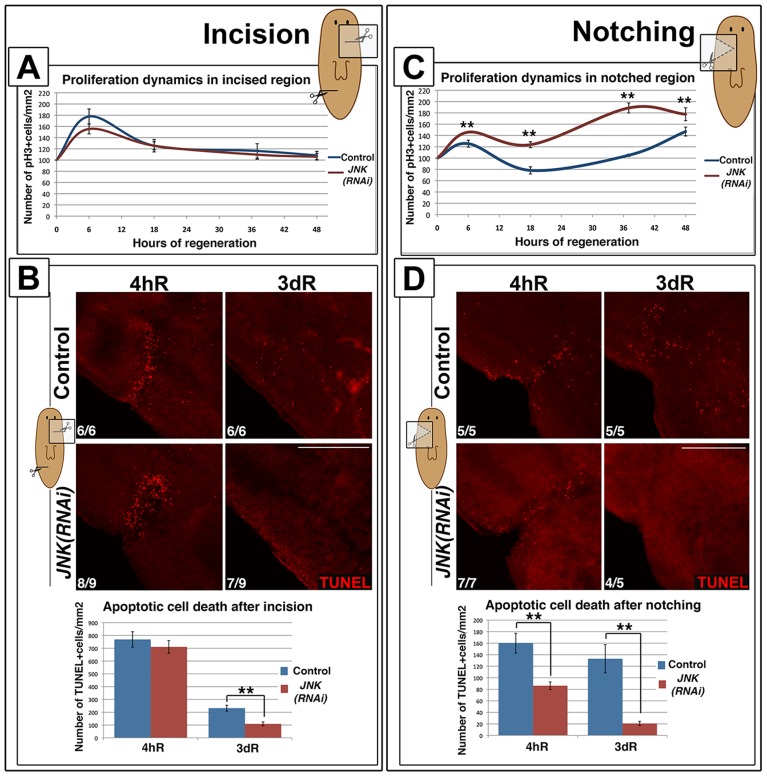
JNK is specifically required for *de novo* tissue regeneration. (**A**) Graph showing proliferation dynamics after a lateral incision without loss of tissue. At least four biological replicates (2 incisions per organism) were used per time point. (**B**) Whole-mount TUNEL staining showing apoptotic cell death responses and graph depicting the quantification of TUNEL-positive cells after a simple incision. At least six biological replicates (2 incisions per organism) were used per time point. (**C**) Graph showing proliferation dynamics after a small lateral amputation with loss of tissue. At least four biological replicates were used per time point for quantification. (**D**) Whole-mount TUNEL staining showing apoptotic cell death responses and graph depicting the quantification of TUNEL-positive cells after a small lateral amputation. At least five biological replicates were used per time point. (Top left, anterior). TUNEL images correspond to confocal z-projections. The area analyzed for quantification of TUNEL+ cells density was more restricted to the wound region for incisions than for notchings. Error bars represent the standard error of the mean. Data were analyzed using a Student's t-test. **P<0.01; differences are considered significant at P<0.05. Scale bars: 200 µm. hR, hours of regeneration; dR, days of regeneration.

Wound signaling induced by a simple incision without the loss of tissue causes cell responses different from that induced by amputation [19]. Healing of a simple incision is characterized by single apoptotic and mitotic peaks; secondary peaks are only observed in response to injury involving tissue loss [19] (Figure 5A-5B-S8A). Wound-associated mitotic and apoptotic responses after a simple lateral incision did not differ between *JNK(RNAi)* and control animals (Figure 5A-5B-S8A). Moreover, the expression of early response genes, normally triggered by this type of injury [15], was unchanged in *JNK(RNAi)* animals (Figure S8B).

To determine the degree of injury that requires JNK-mediated regeneration we performed lateral amputations, removing small portions of tissue (notching). As seen after complete amputation, we observed two temporally coordinated mitotic peaks in control animals (Figure 5C-S8C). In *JNK(RNAi)* animals, both proliferative peaks were affected in the same manner observed after anterior amputations: the first mitotic peak was augmented and the second occurred 10 hours earlier than in controls (Figure 5C-S8C). Analysis of the apoptotic rates in control animals revealed that lateral notches also induced two apoptotic responses: an early wound-related peak and a second more systemic peak, reflecting the need for remodeling after the loss of tissue. As observed after anterior amputation, JNK was essential to trigger an apoptotic response after notching (Figure 5D) and regeneration was impaired in *JNK(RNAi)* animals, as evidenced by the disrupted ventral nerve cord (Figure S8D). Thus, in contrast to the healing process observed after simple incision, regeneration after notching requires JNK for proper cell cycle dynamics and apoptosis. Taken together, these findings demonstrate that JNK is only essential for responses that require the regeneration of missing tissues.

### Rescaling during degrowth requires JNK-dependent activation of apoptosis

**Figure 6:**
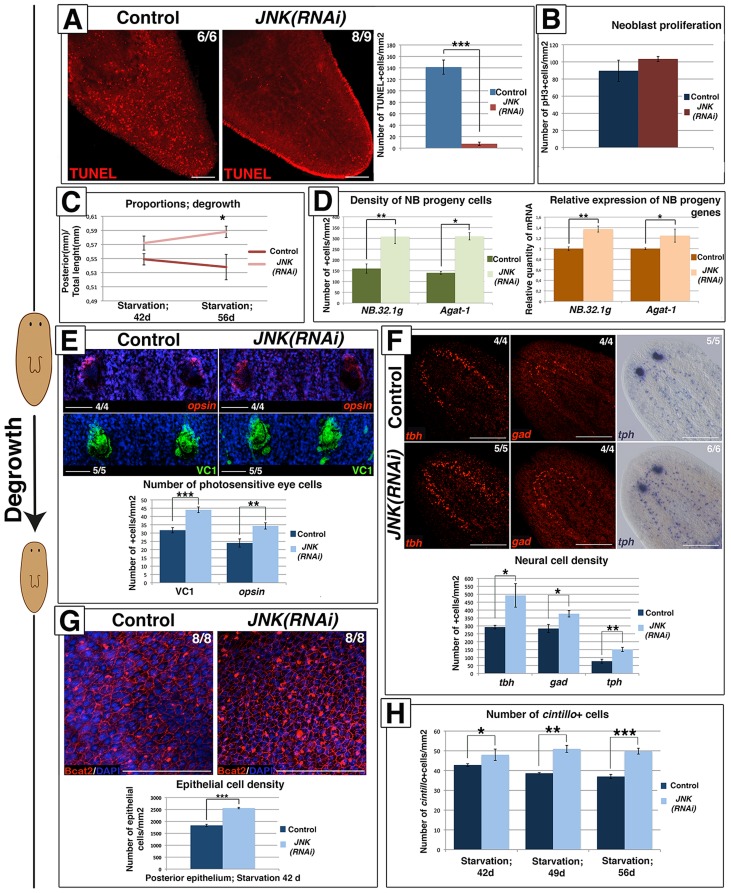
JNK-dependent apoptosis is required for proper remodeling during degrowth. (**A**) Whole-mount TUNEL staining showing apoptotic cell death during degrowth in post-pharyngeal regions and graph showing quantification of cells dying by apoptosis (TUNEL-positive) in animals starved for 42 days. At least six biological replicates were used. (**B**) Graph showing quantification of mitotic (pH3+) cells in animals starved for 42 days. At least nine biological replicates were used. (**C**) Graph showing the size of the post-pharyngeal region (from pharynx anchoring to tail tip) relative to the whole-body length during degrowth. Values represent the means of 15 biological replicates. (**D**) Quantification of *NB.32.1g* and *Agat-1* expression in starved animals. The green histograms depict the quantification of cells positive for these two markers after FISH. At least four biological replicates were used. The orange histograms depict the relative expression of the two markers as determined by qRT-PCR. Values represent the means of three biological replicates. (**E**). FISH analysis of photoreceptor cells (*opsin*+), immunostaining of the visual system (VC1+) with DAPI counterstaining and graph showing the quantification of the number of photoreceptor cells/eye in animals starved for 42 days. Four biological replicates (8 eyes) were used. (**F**) FISH analysis of GABAergic (*gad*+) and octopaminergic (*tbh*+) neurons, WISH analysis of serotoninergic (*tph*+) neurons and graph showing the quantification of the number of neural cells/mm^2^ in the brains of animals starved for 42 days. (**G**) Staining of the epithelia with DAPI and anti-β-catenin-2 antibody (Bcat2), and graph showing the quantification of the number of epithelial cells/mm^2^ in post-pharyngeal regions of animals starved for 42 days. Eight biological replicates were used. (**H**) Quantification of the number of *cintillo*+ cells in the anterior region during the starvation process. At least five biological replicates were used. (Top left, anterior). All images, except for the *tph* image, correspond to confocal z-projections. Error bars represent standard error of the mean. Data were analyzed by Student's t-test. *P<0.05; **P<0.01; ***P<0.001; differences are considered significant at P<0.05. Scale bars: 100 µm (A), 50 µm (E), 300 µm (F), 50 µm (G). d, days of starvation.

Planarians undergo degrowth in the absence of nutrients while maintaining their physiological functions [8]. Degrowth is accompanied by an increase in cell death [17], [41] and maintenance of the neoblast population and baseline rates of proliferation. During starvation-induced degrowth (Figure S9A–S9B) *JNK* RNAi resulted in the near to complete abolition of apoptotic cell death (Figure 6A). However, baseline mitotic rates and neoblast population levels remained constant after JNK RNAi (Figure 6B-S9C-S9D).

Planarians exhibit allometric rescaling during their continuous size changes with positive rescaling of the post-pharyngeal region as compared with the pre-pharyngeal region [42]. We investigated whether the imbalance between apoptosis and proliferation in *JNK(RNAi)* animals undergoing degrowth altered rescaling. To this end, we analyzed proportionality by measuring the length of the posterior portion of the body (from the pharynx anchoring to the tail tip) relative to whole-body length (Figure S9E). No decrease in the length of the posterior portion was observed in *JNK(RNAi)* versus control animals as starving proceeded (42 days vs. 56 days of starvation), indicating that rescaling is impaired when JNK-dependent apoptosis is prevented (Figure 6C). Starvation-induced degrowth leads to a decrease in the number of neoblast progeny cells [41] and a coordinated reduction in the number of cells in pre-existing organs to accommodate to the adjustment in body size. We studied the effects of blocking apoptosis in degrowing *JNK(RNAi)* animals on the number of progeny cells and the morphogenesis of structures such as the epithelium, eyes and brain. The decrease in neoblast progeny observed in *JNK(RNAi)* animals was less pronounced than that seen in starved control animals (Figure 6D-S9F). Quantification of the number of visual system cells (VC1+, *opsin*+) [29], [43], specific neuronal cell types (GABAergic, *gad*+ [44]; octopaminergic, *tbh*+ [45] and serotoninergic, *tph*+ [46]) and epithelial cells, revealed an increase in the density of all differentiated cell types tested in degrowing *JNK(RNAi)* animals (Figure 6E-6F-6G-S9G). Moreover, analysis of the population of *cintillo*+ cells during starvation revealed a decreasing number of this anterior-specific cell type in degrowing control animals but not in degrowing *JNK(RNAi)* animals (Figure 6H-S9H). Analysis of brain morphology in DAPI-stained tissue sections indicated that the increase in cell density in *JNK(RNAi)* animals was associated with a disruption of brain architecture (Figure S9I).

**Figure 7:**
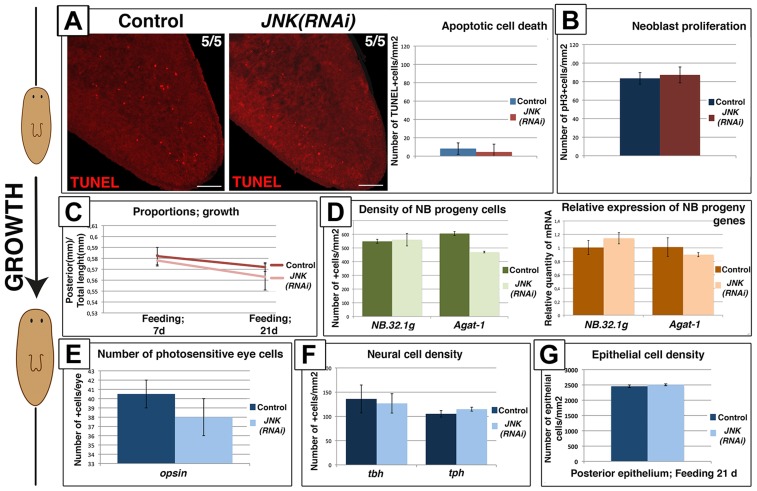
Remodeling during growth does not depend on JNK-dependent apoptotic cell death. (**A**) Whole-mount TUNEL staining showing apoptotic cell death during growth in post-pharyngeal regions and graph showing the quantification of cells dying by apoptosis (TUNEL+) in animals fed for 7 days. At least five biological replicates were used. (**B**) Graph showing quantification of mitotic cells (pH3+) in animals fed for 7 days. At least 10 biological replicates were used. (**C**) Graph showing the size of the post-pharyngeal area (from the pharynx anchoring to tail tip) relative to whole-body length during growth. Values represent the means of 15 biological replicates. (**D**) Quantification of *NB.32.1g* and *Agat-1* expression. Green histograms depict the quantification of cells positive for these two markers after FISH. At least four biological replicates were used. Orange histograms depict the relative expression levels of the two markers as determined by qRT-PCR. Values represent means of three biological replicates. (**E**) Quantification of the number of photoreceptor cells (*opsin*+)/eye of animals fed for 21 days. Three biological replicates (6 eyes) were used. (**F**) Quantification of the number of neural cells/mm^2^ in the brains of animals fed for 21 days. At least four biological replicates were used (**G**) Quantification of the number of epithelial cells/mm^2^ in post-pharyngeal regions of animals fed for 21 days. Six biological replicates were used. (Top left, anterior). All images correspond to confocal z-projections. Error bars represent standard error of the mean. Data were analyzed by Student's t-test. Differences are considered significant at P<0.05. Scale bar: 100 µm. d, days of feeding.

The process of degrowth is reversible; starved planarians return to their original size when feeding is reinstated [8], [41]. A proliferative peak has been described 1 day after feeding, after which mitotic rates return to baseline levels [38], [41]. Planarian growth is also accompanied by a decrease in apoptotic cell death to minimum levels [17] and an increase in the number of neoblast progeny cells [41]. During growth promoted by sustained feeding (Figure S10A–S10B), *JNK(RNAi)* animals exhibited no changes in the rate of apoptotic cell death since minimal levels of apoptosis were also observed in growing control animals (Figure 7A). Furthermore, growing *JNK(RNAi)* maintained neoblast population levels and baseline mitotic rates (Figure 7B-S10C–S10D). In agreement with these observations, growing *JNK(RNAi)* planarians underwent normal rescaling (Figure 7C-S10E). Moreover, we observed no differences in the number of neoblast progeny cells (Figure 7D-S10F), density of the differentiated cells analyzed (Figure 7E-7F-7G-S10G-S10H-S10I) or in brain structure (Figure S10J) between growing *JNK(RNAi)* animals and controls.

Taken together, these findings demonstrate that JNK is required to maintain body proportions and to remodel organs by the induction of apoptotic cell death specifically during degrowth when apoptosis is required to allow size reduction.

## Discussion

### JNK-mediated control of cell cycle progression is required for the stem cell proliferative response during planarian regeneration

The coordination of cell proliferation, differentiation, and apoptosis is central to a number of physiological and pathological processes, including tissue homeostasis, development and cancer [47]. Planarians are a promising model organism for the *in vivo* analysis of the coordination of these processes. The molecular basis for the initiation of neoblast proliferation in response to injury remains largely unknown. It has been proposed that the first mitotic peak would correspond to the shortening of the G2/M transition of neoblasts that were in G2- or S-phase at the time of the injury [18], [19]. Furthermore, given that the mitotic minimum between the two peaks is not due to the cessation of proliferative input, it has been proposed that the same early wound-induced signal triggers the two temporally distinct mitotic peaks [19]. These findings suggest that the two proliferative waves correspond to different sets of neoblasts: those in the G2- or S-phase that enter mitosis earlier and produce the first mitotic peak, and those still in G0/G1-phase that produce the second one, as they need to migrate and complete the cell cycle to enter mitosis [18]. Neoblast descendants from the first peak may also participate in the second peak. Our CldU labeling results showed that 6 hours after amputation, all pH3 positive cells were also positive for CldU (pH3+/CldU+), indicating that all the cells entering mitosis 6 hours post-amputation had been in S-phase during CldU labeling. The fact that we were unable to detect neoblasts in the G2-phase prior to amputation (pH3+/CldU-) supports the hypothesis that this G2 subpopulation does not significantly contribute to the first proliferative peak [48]. However, two factors must be considered: i) these neoblasts would be the first ones reaching M-phase earlier since the first mitotic response already starts 3 hours after cutting [19], [37], and ii) these neoblasts might have already started to enter mitosis during the previous stimulus induced by the CldU injection (10 hours before amputation).

The role of JNK as a cell cycle checkpoint has been previously described in human cell cultures. JNK responds to stress stimuli by delaying the G1/S [49] and the G2/M transitions, thus temporally controlling the onset of mitosis [22], [23]. Interestingly, our double CldU/pH3 labeling results showed an increase in mitotic cells in *JNK(RNAi)* animals while maintaining the number of cycling cells that have gone through S-phase. These results indicate that in planarians JNK attenuates cell cycle progression between G2- to M-phase rather than between G1- to S-phase transition.

The sharper and earlier second mitotic peak seen in *JNK(RNAi)* versus control animals further supports compression of the cell cycle progression of neoblasts after JNK inhibition. The increase of mitotic neoblasts in *JNK(RNAi)* animals five days after anterior amputation may indicate that the mitotic response remains active due to failed execution of the regenerative response.

A previous study on the role of JNK in the planarian species Dugesia japonica showed that treatment with the JNK inhibitor SP600125 blocks the entry of neoblasts into M-phase, leading the authors to propose a role for JNK in promoting the G2- to M-phase transition of neoblasts [50]. The lack of consistency between those findings and our own data might be due to species-specific differences or off-target effects by SP600125, which can bind to a broad range of protein kinases [51], [52].

Our data reveal a molecular mechanism in planarians that controls the onset of neoblast division in response to injury, specifically by the attenuation of the G2- to M transition.

### JNK controls wound signaling, cell death and coordinated stem cell proliferation specifically elicited by tissue loss

**Figure 8:**
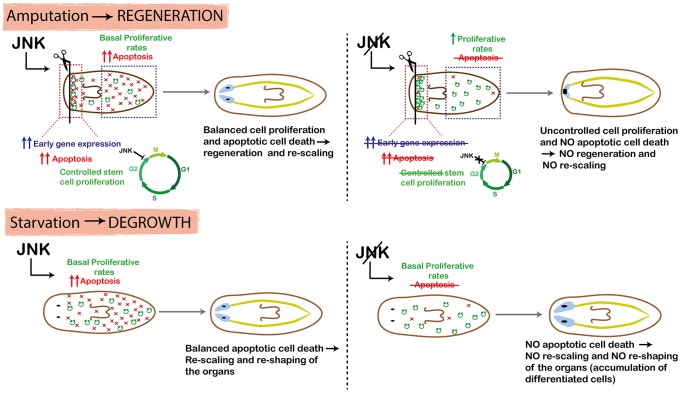
Schematic showing role of JNK in planarian regeneration and homeostatic degrowth. In the wound region, JNK triggers early-gene expression and apoptosis, and mediates temporal control of the cell cycle progression of neoblasts, which ensures the balanced differentiation of different cell types and hence proper regeneration of missing tissues. In pre-existing regions, JNK triggers apoptosis and maintains basal levels of proliferation to ensure that body proportion is properly restored after amputation. RNA interference of JNK activity prevents all these processes in both the wound region and in pre-existing regions, as well as regeneration and rescaling.

Injury triggers a plethora of signals that regulate the onset of regeneration. In planarians, early regeneration involves not only temporally controlled neoblast proliferation [18], [19], but also the induction of the expression of a repertoire of early genes [15], [16] and an apoptotic response [17], accompanied by tightly coordinated cell differentiation and identity specification (Figure 8). Our results demonstrate that in contrast to the previously described function of JNK during *Drosophila* imaginal disc regeneration [53], [54], wound closure in planarians is JNK-independent, probably because in planarians this is a predominantly mechanical process that does not require directed cell migration through actin cables and filopodial extensions. However, JNK is required during the first stages of regeneration to properly induce the expression of early wound-induced genes, the initiation of the wound-associated apoptotic peak and the controlled onset of mitosis (Figure 8). Due to the pleiotropy of JNK, we are not able to discern if JNK independently controls all these processes, or if they are functionally dependent.

Both the wound-related expression of *Runt1*, which has been directly linked with the specification of neural precursors [16], and the production of *ovo*+ eye progenitors [28] were decreased after amputation in *JNK(RNAi)* animals as compared with controls. This decrease points to impaired production of neural progenitor cell types, as also suggested by the impaired regeneration of neural tissues in these animals. Regeneration after notching, a process that does not require anterior or posterior re-specification, was also defective in *JNK(RNAi)* animals. Given that early establishment of polarity was unaffected by *JNK* RNAi, these findings suggest that JNK is required for tissue regeneration of structures and the production of neural progenitor cells, independently of the initial identity specification.

Expression of the post-mitotic neoblast progeny markers *NB.32.1g* and *Agat-1* is associated with the initiation of the differentiation process [39]. The maintenance of their expression in *JNK(RNAi)* planarians suggests that, although the cell cycle is altered, the neoblasts can exit their proliferative cycle and produce the progeny related to these specific markers. Alternatively, a reduction in the number of progeny cells related to defective differentiation in *JNK(RNAi)* animals might be compensated for the lack of death of these progeny cells, making the reduction impossible to detect. However, the nature of the cells expressing these progeny genes remains obscure. It has been shown that these genes do not specify a common state for all post-mitotic neoblasts [55], and there is no evidence indicating that they mark a specific cell lineage.

Whether induction of apoptosis or the control of mitotic onset is the most relevant JNK function during regeneration remains to be elucidated. On the one hand, several recent findings support a link between alterations in cell cycle progression and improper differentiation; shortening of the cell cycle can inhibit neuronal differentiation during *Xenopus* development [56] and enhance the differentiation of epidermal keratinocytes [57]. In planarians, JNK-mediated arrest of cell cycle progression may be required to provide a time window during which neoblasts embark upon specific programs of differentiation. In that sense, our data support the recently proposed “specialized” neoblast model [19], [58], which proposes that a pluripotent neoblast commences determination before becoming post-mitotic progeny, giving rise to specialized progenitors. On the other hand, wound-induced apoptosis has been shown to be essential for tissue regeneration since apoptotic cells release mitogenic factors to stimulate the proliferation of surrounding cells, a process known as compensatory proliferation [47], [59], [60], which has been recently renamed “apoptosis-induced proliferation” [61]. Our results, however, indicate that apoptosis-induced proliferation might not occur during planarian regeneration. While *JNK* RNAi led to a reduction in apoptosis, a subsequent reduction in proliferation was not observed either in the blastema or during the remodeling of pre-existing tissues. The induction of neoblast proliferation in response to amputation or during tissue turnover thus appears to be independent of factors released by apoptotic cells, if present. Alternatively, given that compensatory proliferation is controlled by JNK during *Drosophila* wing disc development [62], it is possible that no direct relationship between apoptosis and proliferation is observed in *JNK(RNAi)* animals because compensatory proliferation may also be JNK-dependent in planarians. Furthermore, it should be noted that JNK is required for the appropriate expression of the early-wound induced genes, probably also essential to launch regeneration [15], [16].

Finally, our data demonstrate that JNK is required for appropriate regenerative responses elicited exclusively by tissue loss and not after simple wounding. Therefore, in agreement with previous studies demonstrating distinct roles of the two mitotic peaks (at 6 h and 48 h) depending on the degree of injury [19] and the requirement of *Smed-follistatin* specifically for the “missing-tissue response” [63], our findings distinguish between the response to a small incision and that of an amputation. According to the role of JNK as a stress-activated protein to coordinate a complex response, JNK is exclusively required in large scale tissue recovery events, whereas mild tissue damage can be repaired in a JNK-independent manner.

### JNK-dependent apoptosis is required for remodeling and the maintenance of body proportion

The ability to generate a proportioned organism after amputation depends on a carefully maintained balance between stem cell proliferation and differentiation to create new tissues, and cell death to eliminate unnecessary cells and re-shape organs [47]. Planarians respond to amputation by increasing neoblast proliferation and differentiation in regions close to the wound in order to regenerate the missing structures. In parallel, pre-existing regions decrease in size in order to adjust their proportions relative to the new whole-body size, mainly by augmenting cell death while maintaining baseline proliferative rates [17], [41], [64]–[66] (Figure 8). Similarly, proliferative rates are maintained during homeostatic changes in body size, whereas the control of cell death is the decisive shift. Our findings demonstrate that JNK is essential to trigger apoptotic cell death in planarians. In agreement with the essential role of apoptosis during planarian remodeling [17], [40], animals in which JNK-mediated apoptotic cell death was prevented were unable to relocate pre-existing structures and hence to restore body proportion not only after amputation, but also during starvation-induced degrowth (Figure 8). The pro-apoptotic role of JNK in vertebrates, in which JNK acts as a tumor suppressor gene, has been extensively documented [20]. Our results show that after *JNK* RNAi, the positive balance between cell death and cell proliferation required to decrease body size is reversed (Figure 8). However, we observed no overgrowths in *JNK(RNAi)* animals. A tempting explanation is that the lack of apoptosis results in an ectopic accumulation of certain cell types, as reflected by the increased density of differentiated cells, such as neural and epithelial cells, observed in degrowing *JNK(RNAi)* planarians.

*JNK* RNAi during diet-induced changes in body size resulted in impaired rescaling only in planarians that were starved, and hence decreased in size. By contrast, animals that were growing while actively feeding exhibited normal body proportions. While downregulation of apoptosis is essential during active feeding so that planarians can grow, high rates of apoptosis are required during starvation. Our results show that JNK-dependent apoptosis is only required during degrowth, while proliferation is maintained during both conditions. These findings demonstrate that defective rescaling and the accumulation of neoblast progeny and differentiated cells in degrowing *JNK(RNAi)* planarians are a consequence of an inhibited apoptotic response. Remarkably, the number of *cintillo*+ cells was maintained during starvation in *JNK(RNAi)* animals, indicating that differentiated cells accumulated due to the lack of cell death, and not as a result of ectopic cell differentiation. Finally, our data indicate that apoptotic cell death is required during homeostatic tissue turnover not only to re-scale but also to re-shape organs, as the ectopic accumulation of differentiated cells in JNK(RNAi) animals might be associated with ultrastructural changes in the brain.

That the maintenance of mitotic rates during gradual degrowth is independent of JNK supports the role of JNK as a molecular modulator of cell cycle progression specifically during regeneration, a process that requires the coordination of massive neoblast proliferation and differentiation. Similar to planarians, *Drosophila* imaginal discs undergo dynamic regulation of cell death and proliferation, as growth depletion in one compartment of the *Drosophila* wing disc results in increased apoptotic cell death and decreased proliferation in the adjacent compartment, reducing its size in order to maintain a properly proportioned organ [67]. It has been proposed that the combined autonomous and non-autonomous activity of p53 is fundamental in this process, as it exerts distinct effects in damaged and distant tissues. The generation of differential outputs from a single stress response element, based on proximity to the injury, may be a general mechanism to coordinate basic cellular responses in order to maintain proportionality during regeneration.

## Materials and Methods

### Planarian culture

Planarians used in these experiments were from a clonal strain of the *S. Mediterranea* BCN-10 biotype and were maintained as previously described [68]. Planarians were starved for 1 week and were 4 to 6 mm in length when used for experiments.

### Isolation of *Smed-JNK*

*Smed-JNK* fragments were identified from the *S. mediterranea* genomic contigs (Washington University, St. Louis, USA). The following pairs of specific primers were used to clone a fragment of *Smed-JNK*:

5′-:GCTATTGGTTCCGGTGCACAAG-3′;

5′-:CGACCGAGATTCGTTGAAGTGG-3′

The corresponding full-length transcripts were amplified by rapid amplification of cDNA ends (RACE) using the Invitrogen GeneRacer Kit (Invitrogen).

### RNAi analysis

Double-stranded RNAs (dsRNAs) were synthesized by *in vitro* transcription (Roche) as previously described [43], and dsRNA microinjections performed as previously described [43], following the standard protocol of a 3×32-nl injection of dsRNA for three consecutive days before amputation (one round of injections). To obtain reliable gene interference, we performed three consecutive rounds of RNAi injections; an anterior amputation was performed after the first and third rounds. Control animals were injected with water or a double-stranded (ds) RNA of the green fluorescent protein (GFP) sequence. The same RNAi experimental design was used in all experiments but different types of injuries were induced (incision, notching or amputation), as indicated in the text. For degrowth and growth experiments, animals were starved and injected with RNAi for three weeks. Subsequently, degrowing animals were maintained under starvation conditions while growing animals were fed every second day. The following pairs of specific primers were used to generate the dsRNA target gene:

dsRNA against *Smed-JNK*,

5′-:GCTATTGGTTCCGGTGCACAAG-3′;

5′-: GGACGTCCTTTCGTGATCTAAGTCC-3′

### Quantitative real-time PCR

Total RNA was extracted from a pool of five trunk fragments, six wound region fragments and six post-pharyngeal (pre-existing) region fragments of RNAi-treated planarians using TRIzol reagent (Invitrogen). RNA samples were treated with DNase I (Roche) and cDNA was synthesized using a First-Strand Synthesis System kit (Invitrogen). Real-time PCR was performed using SYBR Green (Applied Biosystems) in an ABI Prism 7900HT Sequence Detection System (Applied Biosystems). Three samples were run in parallel for each condition. Data were normalized to the expression of the internal control (UDP). Similar results were obtained using elongation factor 2 (EF-2) as an alternative internal control. The following sets of specific primers were used:

*Smed-JNK* mRNA,

5′-TCAACGAATCTCGGTCG-3′, 5′-AGTGAGCTCTCTTTCATCAACC-3′;

*Smed-h2b* mRNA,

5′-GAGAAAGTTGAACGGCCC-3′, 5′-AAGATAATACGTACTTCAACGACG-3′;

*Smed-Agat-1* mRNA,

5′-GCCCAGAAAGACCATGC-3′, 5′-GAGACAACCATTGAGAGCTG-3′;

*NB32.1g* mRNA,

5′-CATCGCGCAACTTTTG-3′, 5′-GTTTACGGAGAATGCCG-3′;

*Smed-UDP* mRNA was detected using primers previously described in [69]:

*Smed-EF-2*,

5′-CGAGCCGGAAGATTTGTAT-3′, 5′-TGGAGTCACTTGAATATCTCC-3′.

### Irradiation

Intact planarians were X-irradiated at 96 Gy and fixed for *in situ* hybridization 1 day after irradiation.

### Whole-mount *in situ* hybridization (ISH) and fluorescent whole-mount *in situ* hybridization (FISH)

RNA probes were *in vitro* synthesized using Sp6 or T7 polymerase (Roche) and DIG-, FITC- (Roche) or DNP- (Perkin Elmer) modified ribonucleotides. RNA probes were purified by ethanol precipitation and the addition of 7.5 M ammonium acetate. For ISH, animals were fixed and then processed using an In situ Pro hybridization robot (Abimed/Intavis), as previously described [70]. Hybridizations were carried out for 16 h at 56 C. Samples were observed using a Leica MZ16F microscope and images were captured with a Leica DFC300FX camera. For FISH, animals were fixed and processed as previously described [71]. Confocal laser scanning microscopy was performed using a Leica TCS 4D (Leica Lasertechnik, Heidelberg) adapted for an inverted microscope (Leitz DMIRB). Images were processed using Fiji software [72]. The numbers of *NB32.1g+* and *Agat-1+* cells were quantified using the “Find maxima” plug-in in Fiji, maintaining a fixed noise tolerance of 100 and correcting by hand. *opsin+*, *gad+*, *tbh*+, *tph*+ and *cintillo+* cells were quantified by hand using the “multi-point selection“ tool in Fiji. *egrl-1* and *runt-1* images were captured with identical laser settings: 18 stacks were used to build the z-projection and the intensity of equivalent areas was quantified using the “Measure” plug-in in Fiji.

### Immunostaining

Immunostaining was carried out as described previously [43]. The following antibodies were used: anti-synapsin (anti-SYNORF1, 1∶50; Developmental Studies Hybridoma Bank), anti-Smed-β-catenin2 (1∶1000; Chai et al., 2010) and anti-phospho-histone H3 (Ser10) (D2C8) (pH3) (1∶500; Cell Signaling Technology). Images were scanned, processed and quantified as described for FISH images. To avoid technical variance and obtain a reliable quantification of pH3+ cells, at least two independent experiments of RNAi and pH3 immunostaining were carried out for anterior amputation, incision, degrowth and growth experimental designs.

### Whole-mount TUNEL

Animals were fixed and stained for TUNEL as previously described [17] using the ApopTag Red *In Situ* Apoptosis Detection Kit (CHEMICON, S7165), with some modifications to increase permeability: between the fixation step with 4% formaldehyde in PBST, samples were incubated with ProteinaseK (20 µg/mL) in PBSTx (PBS with 0.3% Triton X-100) for 10 minutes at 37°C in a water bath while agitating by hand, and an additional reduction step was added after fixation [71]. Finally, samples were incubated overnight in terminal transferase enzyme at 37°C, and again overnight at 4°C with anti-dioxigenin-rhodamine. Images were scanned, processed and quantified as described for FISH images. To avoid technical variance and obtain a reliable quantification of TUNEL+ cells, at least two independent experiments of TUNEL staining were carried out for anterior amputation, notching, incision, degrowth and growth experimental designs.

### TUNEL in paraffin-embedded tissue

Animals were sacrificed in 10% n-acetyl cysteine in PBS, docked for 8 minutes at RT to remove mucous and fixed in 4% paraformaldehyde in PBS for 4 hours at 4°C. Paraffin embedding, sectioning and de-paraffinization were carried out as previously described [73]. Staining was performed according to the manufacturer's recommendations with the following modifications: sections were treated with ProteinaseK for 30 minutes and with TdT for 2 hours and were incubated in the anti-digoxigenin conjugate overnight at 4°C in a humidified chamber.

### Counting of mitotic cells

Fixation and partial maceration of the animals, staining of nuclei and calculation of the mitotic index were carried out following a modified Gomori technique as previously described [18].

### Flow cytometry

The dissociation of planarians, cellular labeling, and isolation of cells by FACS were performed as described previously [36].

### CldU labeling

After RNAi injection protocol, control and JNK(RNAi) animals were injected a single time 10 hours prior amputation as previously described in [74]. 6 hours after amputation animals were fixed as previously described in [74] for FISH with Smedwi-1 DIG probe. After FISH staining, animals were processed for anti-pH3 immunostaining as described above. Animals were then processed for CldU staining as previously described in [74]. Blocking and incubation with the CldU antibody were performed in 1%BSA/10%NGS in PBSTx.

## Supporting Information

Figure S1 (A) Alignment of Smed-JNK protein. JNK protein sequences of species from the main phylogenetic groups were used. The alignment was processed using MAFFT version 6 (http://mafft.cbrc.jp/alignment/server/index.html). (The submission ID in NCBI of *Smed-JNK* corresponding to the complete mRNA coding sequence is 1620068). Abbreviations: Dm, *Drosophila melanogaster*; Mm, *Mus musculus*; Sc, *Saccoglossus kowalevskii*; Sm, *Schistosoma mansoni*; Smed, *Schmidtea mediterranea*; Tc, *Tribolium castaneum.* (B) Cartoon illustrating the experimental design of *JNK* interference during anterior regeneration. Animals were starved and injected with the RNAi for three weeks to achieve reliable gene interference. Two anterior amputations were performed, one after the first round of RNAi injections and another after the final round of injections. Animals were subsequently allowed to regenerate and then fixed at different time points. (C) Graph showing the relative expression of *JNK* in regenerating animals as determined by qRT-PCR. *JNK* expression levels in *JNK(RNAi)* animals were significantly reduced as compared with controls, validating the gene interference approach. Values represent the means of three biological replicates. Error bars represent standard error of the mean. Data were analyzed by Student's t-test. **P<0.01. dR, days of regeneration.
(TIF)

Figure S2 Initial identity specification during regeneration is independent of JNK. WISH analysis of the expression of the polarity genes *notum*, *sFRP1* and *wnt1* in the wound region in regenerating trunks and heads after anterior amputation. (Top, anterior). Scale bars: 200 µm. hR, hours of regeneration, dR, days of regeneration.
(TIF)

Figure S3 Wound closure in planarians is independent of JNK activity. Stereomicroscopic view of live animals showing normal wound closure even after *JNK(RNA)*. The wound is closed by muscle contraction 5 minutes after amputation. However, after 3 days of regeneration, the shape and size of the blastema in *JNK(RNAi)* animals differed to that of controls. All images correspond to regenerating trunk fragments after a bipolar amputation. (Left, anterior). Scale bar: 300 µm. minR, minutes of regeneration; hR, hours of regeneration; dR, days of regeneration.
(TIF)

Figure S4 Expression of early wound-induced genes is reduced in *JNK(RNAi)* animals. WISH analysis of *egrl1* and *runt1* expression in trunk fragments after anterior amputation. Representative animals of milder *JNK(RNAi)* phenotypes have been placed before than stronger phenotypes. Scoring of the different phenotypes is shown. (Left, anterior). Scale bars: 200 µm. minR, minutes of regeneration; hR, hours of regeneration.
(TIF)

Figure S5 JNK controls cell cycle dynamics in neoblasts but does not maintain cell viability. (A) WISH analysis of the expression of *JNK* and *piwi1* in control animals and after ablation of neoblasts by *h2b* RNAi. (Left, anterior). (B) Expression of *JNK* in untreated animals and in those fixed 1 day after a 96-Gy irradiation. (Left, anterior). (C) Anti-pH3 immunostaining showing the dynamics of the mitotic response in regenerating trunk fragments after anterior amputation. (Top left, anterior). (D) Anti-pH3 immunostaining showing the dynamics of the mitotic response in regenerating trunk fragments after posterior amputation and a graph showing the number of mitotic (pH3+) cells in the wound region of regenerating trunk fragments after posterior amputation. At least four biological replicates were used per time point. (Top left, anterior). (E) Quantification of the number of mitotic cells using a modified Gomori technique and quantification of the number of pH3+ cells in the wound region of regenerating trunk fragments at the same time points after anterior amputation. Gomori Mitotic Index represents the number of mitotic figures observed in 100 cells. (F) Whole-mount fluorescent *in situ* hybridization (FISH) showing the expression of *h2b*, a neoblast-related gene, in regenerating trunk fragments after anterior amputation. (Top left, anterior). (G) Graph showing the relative expression of *h2b*, as determined by qRT-PCR, in regenerating trunk fragments. Values represent the means of three biological replicates. (H) Fluorescence-associated cell sorting (FACS) analysis showing the proportion of distinct cell populations at different time points during regeneration. Values represent the means of at least two biological replicates. The pH3 and *h2b* images correspond to confocal z-projections. Error bars represent the standard error of the mean. Data were analyzed by Student's t-test. *P<0.05; **P<0.01; differences are considered significant at P<0.05. Scale bars: 300 µm. hR, hours of regeneration; dR, days of regeneration; dPI, days post-irradiation.
(TIF)

Figure S6 The numbers of early and late neoblast progeny cells are maintained after JNK RNAi in both regenerating and pre-existing regions. (A) FISH showing the expression of *NB.32.1g*, a marker of early post-mitotic descendants of neoblasts, in regenerating trunk fragments after anterior amputation. Images of the wound and the post-pharyngeal (pre-existing) region are shown. The green histogram depicts the quantification of *NB.32.1g*+ cells after FISH. At least four biological replicates were used per time point. The orange histogram depicts the relative expression of *NB.32.1g* as determined by qRT-PCR. Values represent the means of three biological replicates. Analysis from wound and post-pharyngeal (pre-existing) regions are shown. (Top/top left, anterior). (B) FISH showing the expression of *Agat-1*, a marker of late post-mitotic descendants of neoblasts, in regenerating trunk fragments after anterior amputation. Images of the wound and post-pharyngeal (pre-existing) region are shown. The green histogram depicts the quantification of *Agat-1*+ cells after FISH. At least five biological replicates were used per time point. The orange histogram depicts the relative expression of *Agat-1* as determined by qRT-PCR. Values represent the means of three biological replicates. Analysis from wound and post-pharyngeal (pre-existing) regions are shown. (Top/top left, anterior). All images correspond to confocal z-projections. Error bars represent the standard error of the mean. Data were analyzed by Student's t-test. Differences are considered significant at P<0.05. Scale bars: 200 µm. dR, days of regeneration.
(TIF)

Figure S7 JNK plays a general pro-apoptotic role and coordinates the restoration of body proportion after any kind of amputation. (A) Whole-mount TUNEL staining showing apoptotic cell death in regenerating trunk fragments after anterior amputation. Images of the wound and post-pharyngeal (pre-existing) region are shown. (Top/top left, anterior). (B) TUNEL staining in longitudinal tissue sections showing apoptotic cell death in regenerating trunk fragments after anterior amputation. Images of the wound region 4 hours after amputation and of the post-pharyngeal (pre-existing) region 3 days after amputation are shown. (Left, anterior). (C) Graph showing the quantity of mitotic cells (pH3+) in anterior (pre-existing) regions of regenerating trunks after posterior amputation. At least four biological replicates were used per time point. All images correspond to confocal z-projections. Error bars represent the standard error of the mean. Data were analyzed by Student's t-test. *P<0.05; **P<0.01; Differences are considered significant at P<0.05. Scale bars: 200 µm. dR, days of regeneration.
(TIF)

Figure S8 JNK is specifically required for *de novo* formation of new tissue after injury. (A) Anti-pH3 and anti-PIWI-1 immunostaining showing the dynamics of the mitotic response after a simple incision without loss of tissue. (Top left, anterior). (B) Analysis WISH of the genes expressed in response to incision. (Left, anterior). (C) Anti-pH3 immunostaining showing the dynamics of the mitotic response after a small lateral amputation. (Top left, anterior). (**D**) Anti-synapsin immunostaining demonstrating the regeneration of a previously amputated portion of ventral nerve cord (VNC). (Top left, anterior). The pH3/PIWI1, pH3 and Syn images correspond to confocal z-projections. Scale bars: 200 µm (A), 300 µm (B), 200 µm (C), 200 µm (D). hR, hours of regeneration; dR, days of regeneration.
(TIF)

Figure S9 Role of JNK during homeostatic degrowth. (A) Cartoon illustrating the experimental design of the analysis of JNK function during degrowth. Animals were starved and injected with RNAi for three weeks to achieve reliable gene interference and were subsequently fixed after 42 or 56 days of starvation. (B) Graph showing the relative expression of *JNK* as determined by qRT-PCR in degrowing animals. *JNK* expression levels in *JNK (RNAi)* animals were significantly reduced, validating the gene interference approach. Values represent the means of three biological replicates. (C) Anti-pH3 immunostaining showing mitotic activity after 42 days of starvation. (Top left, anterior). (D) FISH showing the expression of *h2b* in animals starved for 42 days and graph showing the relative expression of *h2b* as determined by qRT-PCR in degrowing animals. Values represent the means of three biological replicates. (Top left, anterior). (E) Immunostaining with anti-synapsin to visualize the central nervous system and the pharynx of degrowing animals. The relative length of the posterior region was calculated by measuring the distance from the pharynx-anchoring to the tail tip and dividing this by the whole-body length. The white asterisk indicates the anchoring of the pharynx. (Left, anterior). (F) FISH showing the expression of *NB.32.1g* and *Agat-1*, markers of post-mitotic descendants of neoblasts, in degrowing animals. (Top left, anterior). (G) Graph illustrating the quantification of the number of epithelial cells/mm^2^ in the anterior regions of animals starved for 42 days. Eight biological replicates were used. (Top left, anterior). (H) WISH analysis of the anterior chemoreceptors (*cintillo*+) during starvation. (Top, anterior). (I) DAPI-stained tissue sections showing the brain of animals starved for 42 days. Corresponding images at increased magnification are shown aside. (Left, anterior). All images except for *cintillo* correspond to confocal z-projections. Error bars represent standard error of the mean. Data were analyzed by Student's t-test. *P<0.05; ***P<0.001; Differences are considered significant at P<0.05. Scale bars: 200 µm (C-E), 300 µm (F), 200 µm (G), 300 µm (I), 100 µm (J). d, days of starvation; w, weeks of starvation.
(TIF)

Figure S10 Role of JNK in homeostatic growth. (A) Cartoon illustrating the experimental design of the analysis of JNK function during growth. Animals were starved and injected with the RNAi for three weeks to achieve reliable gene interference. They were subsequently fed once every two days and were then fixed 7 or 21 days after the first feed. (B) Graph showing the relative expression of *JNK* in growing animals as determined by qRT-PCR. *JNK* expression levels in *JNK(RNAi)* animals were significantly reduced as compared with controls, validating the gene interference approach. Values represent the mean of three biological replicates. (C) Anti-pH3 immunostaining showing mitotic activity after 7 days of feeding. (D) FISH showing the expression of *h2b* in animals fed for 7 days and graph showing the relative expression of *h2b* as determined by qRT-PCR in growing animals. Values represent means of three biological replicates. (Top left, anterior). (E) Immunostaining with anti-synapsin to visualize the central nervous system and the pharynx of growing animals. The relative length of the posterior region was calculated by measuring the distance from the anchoring of the pharynx to the tail tip and dividing this by the whole-body length. White asterisk indicates the anchoring of the pharynx. (Left, anterior). (F) FISH showing the expression of *NB.32.1g* and *Agat-1*, markers of post-mitotic descendants of neoblasts, in growing animals. (G) FISH analysis of photoreceptor cells (*opsin*+) counterstained with DAPI (Top, anterior). (H) FISH analysis of octopaminergic (*tbh*+) neurons and WISH analysis of serotoninergic (*tph*+) neurons in the brains of animals fed for 21 days. (Top left, anterior). (I) Staining of the epithelia with DAPI and anti-β-catenin-2 antibody (Bcat2) and graph illustrating the quantification of the number of epithelial cells/mm^2^ in anterior regions from animals fed for 21 days. Six biological replicates were used. (Top left, anterior). (**J**) DAPI staining in tissue sections from the brains of animals fed for 21 days. All the images except for *cintillo* and *tph* correspond to confocal z-projections. Error bars represent the standard error of the mean. Data were analyzed by Student's t-test. ***P<0.001; Differences are considered significant at P<0.05. Scale bars: 200 µm (C-E), 300 µm (F), 200 µm (G), 50 µm (H), 300 µm (I), 50 µm (J), 100 µm (K). d, days of feeding.
(TIF)
