# Correction: JNK Controls the Onset of Mitosis in Planarian Stem Cells and Triggers Apoptotic Cell Death Required for Regeneration and Remodeling

**DOI:** 10.1371/journal.pgen.1005132

**Published:** 2015-04-10

**Authors:** The PLOS Genetics Staff

The second author's name is spelled incorrectly. The correct name is: Xenia Crespo-Yanez. The correct citation is: Almuedo-Castillo M, Crespo-Yanez X, Seebeck F, Bartscherer K, Salò E, et al. (2014) JNK Controls the Onset of Mitosis in Planarian Stem Cells and Triggers Apoptotic Cell Death Required for Regeneration and Remodeling. PLoS Genet 10(6): e1004400. doi:10.1371/journal.pgen.1004400
