# Supplementary figures and images for: JNK Controls the Onset of Mitosis in Planarian Stem Cells and Triggers Apoptotic Cell Death Required for Regeneration and Remodeling
Source: PLoS Genet. 2014 Jun 12;10(6):e1004400. doi: 10.1371/journal.pgen.1004400 (PMC4055413; doi:10.1371/journal.pgen.1004400)

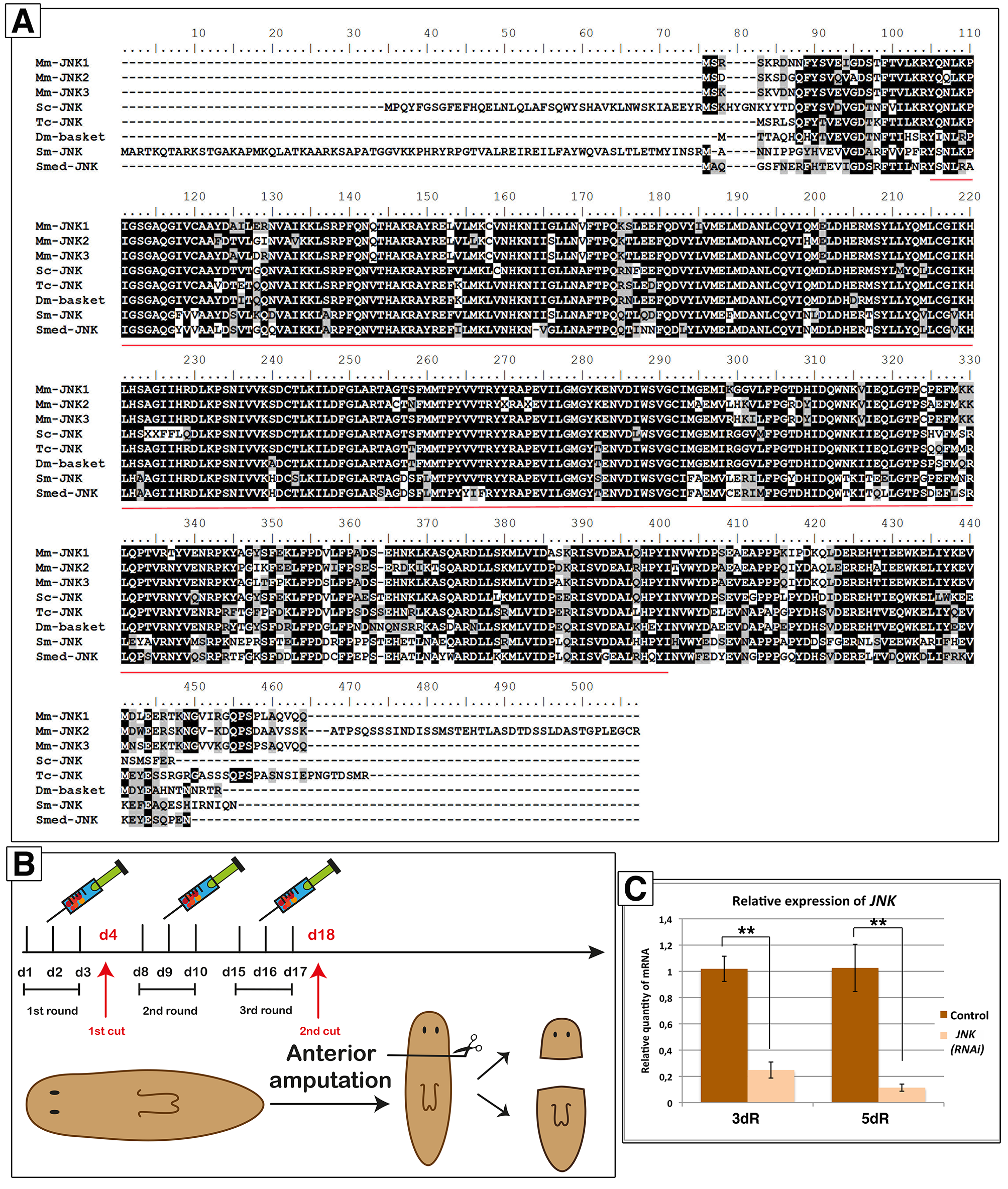

Supplement: Figure S1 — (A) Alignment of Smed-JNK protein. JNK protein sequences of species from the main phylogenetic groups were used. The alignment was processed using MAFFT version 6 (http://mafft.cbrc.jp/alignment/server/index.html). (The submission ID in NCBI of Smed-JNK corresponding to the complete mRNA coding sequence is 1620068). Abbreviations: Dm, Drosophila melanogaster; Mm, Mus musculus; Sc, Saccoglossus kowalevskii; Sm, Schistosoma mansoni; Smed, Schmidtea mediterranea; Tc, Tribolium castaneum. (B) Cartoon illustrating the experimental design of JNK interference during anterior regeneration. Animals were starved and injected with the RNAi for three weeks to achieve reliable gene interference. Two anterior amputations were performed, one after the first round of RNAi injections and another after the final round of injections. Animals were subsequently allowed to regenerate and then fixed at different time points. (C) Graph showing the relative expression of JNK in regenerating animals as determined by qRT-PCR. JNK expression levels in JNK(RNAi) animals were significantly reduced as compared with controls, validating the gene interference approach. Values represent the means of three biological replicates. Error bars represent standard error of the mean. Data were analyzed by Student's t-test. **P<0.01. dR, days of regeneration. (TIF) [file pgen.1004400.s001.tif]

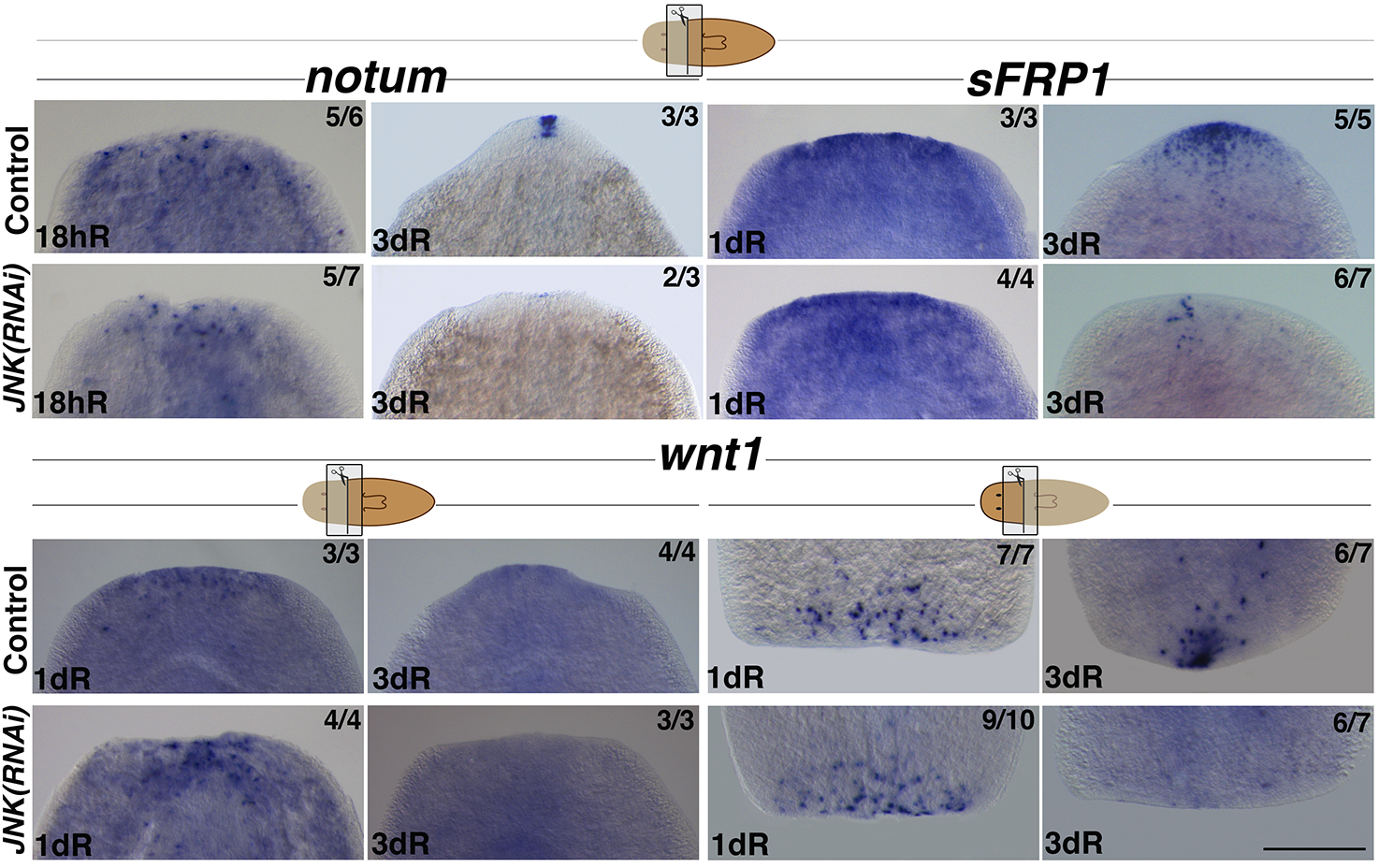

Supplement: Figure S2 — Initial identity specification during regeneration is independent of JNK. WISH analysis of the expression of the polarity genes notum, sFRP1 and wnt1 in the wound region in regenerating trunks and heads after anterior amputation. (Top, anterior). Scale bars: 200 µm. hR, hours of regeneration, dR, days of regeneration. (TIF) [file pgen.1004400.s002.tif]

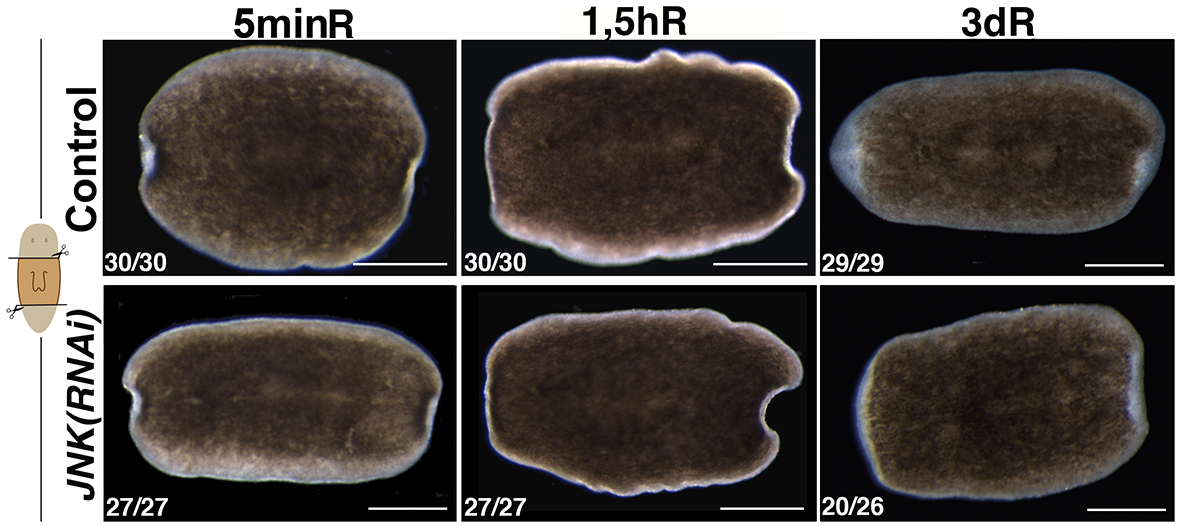

Supplement: Figure S3 — Wound closure in planarians is independent of JNK activity. Stereomicroscopic view of live animals showing normal wound closure even after JNK(RNA). The wound is closed by muscle contraction 5 minutes after amputation. However, after 3 days of regeneration, the shape and size of the blastema in JNK(RNAi) animals differed to that of controls. All images correspond to regenerating trunk fragments after a bipolar amputation. (Left, anterior). Scale bar: 300 µm. minR, minutes of regeneration; hR, hours of regeneration; dR, days of regeneration. (TIF) [file pgen.1004400.s003.tif]

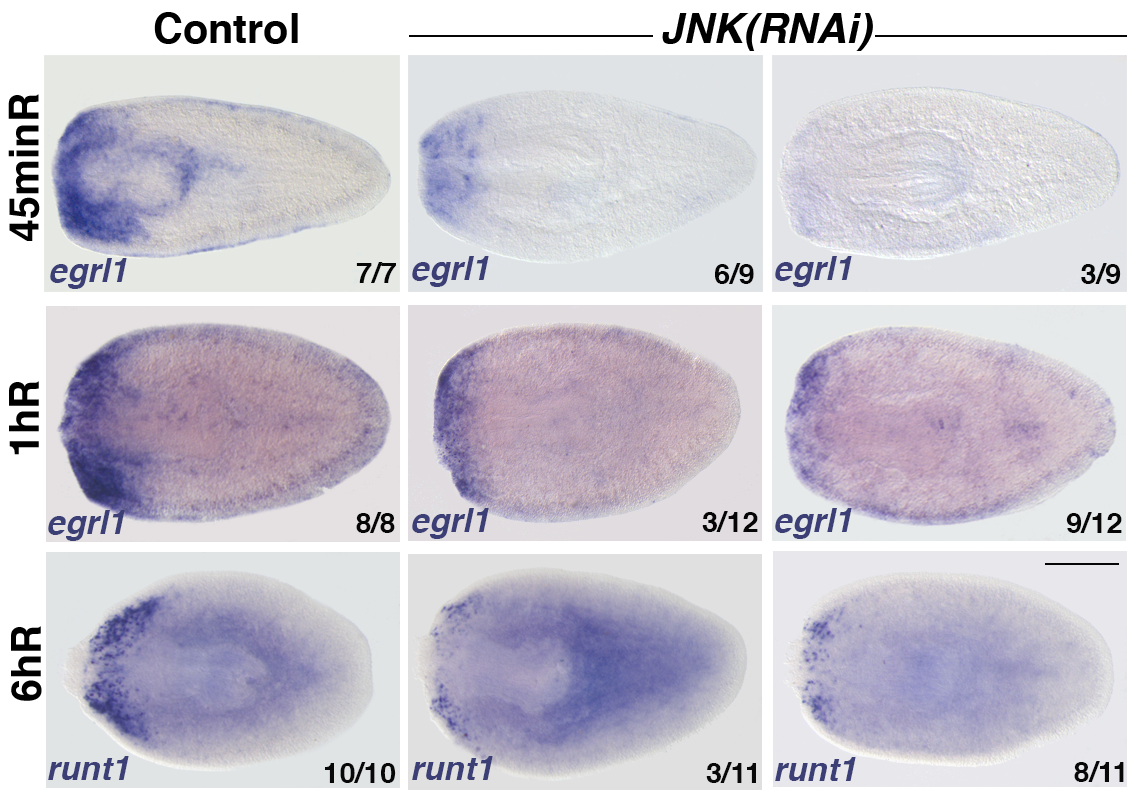

Supplement: Figure S4 — Expression of early wound-induced genes is reduced in JNK(RNAi) animals. WISH analysis of egrl1 and runt1 expression in trunk fragments after anterior amputation. Representative animals of milder JNK(RNAi) phenotypes have been placed before than stronger phenotypes. Scoring of the different phenotypes is shown. (Left, anterior). Scale bars: 200 µm. minR, minutes of regeneration; hR, hours of regeneration. (TIF) [file pgen.1004400.s004.tif]

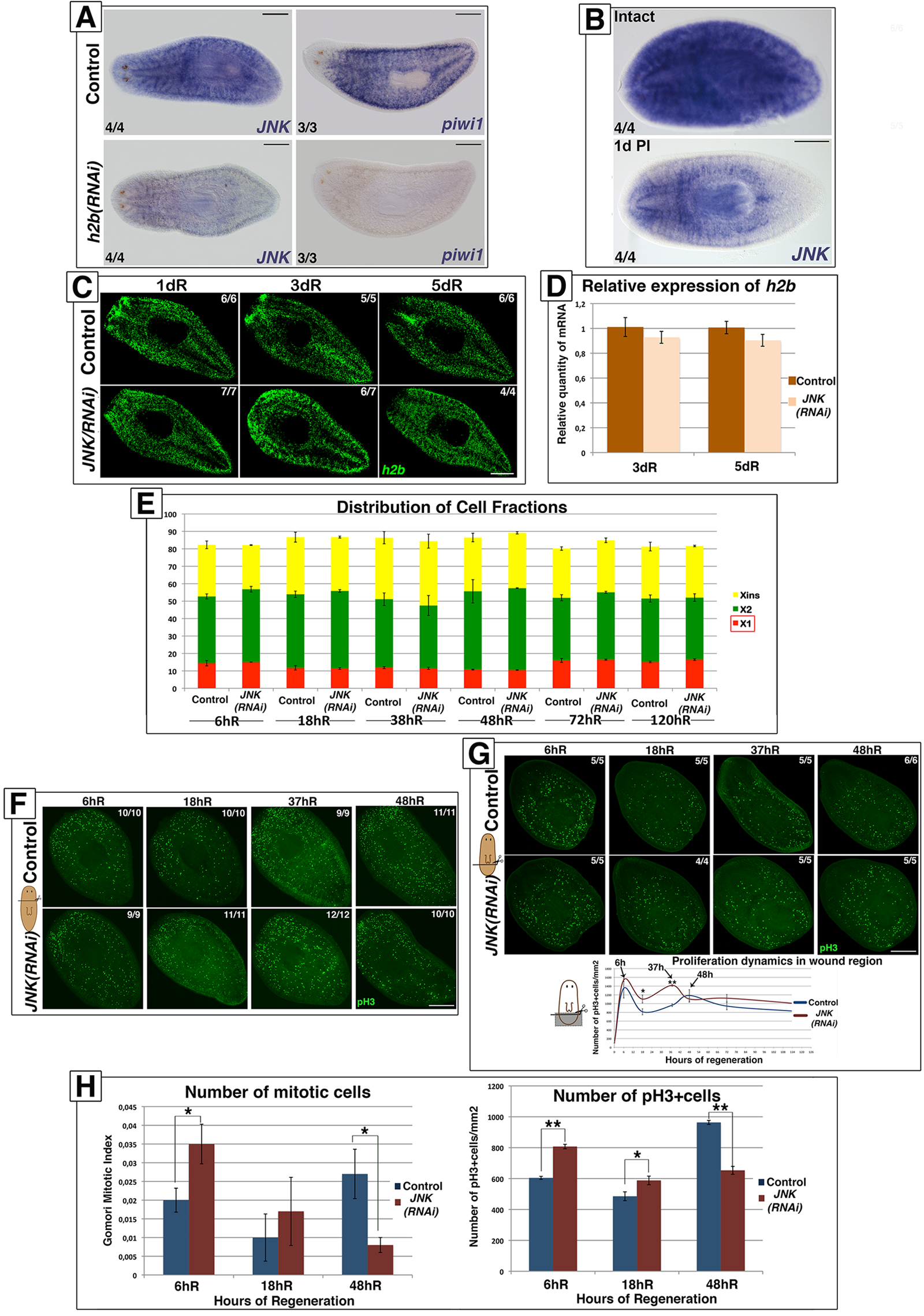

Supplement: Figure S5 — JNK controls cell cycle dynamics in neoblasts but does not maintain cell viability. (A) WISH analysis of the expression of JNK and piwi1 in control animals and after ablation of neoblasts by h2b RNAi. (Left, anterior). (B) Expression of JNK in untreated animals and in those fixed 1 day after a 96-Gy irradiation. (Left, anterior). (C) Anti-pH3 immunostaining showing the dynamics of the mitotic response in regenerating trunk fragments after anterior amputation. (Top left, anterior). (D) Anti-pH3 immunostaining showing the dynamics of the mitotic response in regenerating trunk fragments after posterior amputation and a graph showing the number of mitotic (pH3+) cells in the wound region of regenerating trunk fragments after posterior amputation. At least four biological replicates were used per time point. (Top left, anterior). (E) Quantification of the number of mitotic cells using a modified Gomori technique and quantification of the number of pH3+ cells in the wound region of regenerating trunk fragments at the same time points after anterior amputation. Gomori Mitotic Index represents the number of mitotic figures observed in 100 cells. (F) Whole-mount fluorescent in situ hybridization (FISH) showing the expression of h2b, a neoblast-related gene, in regenerating trunk fragments after anterior amputation. (Top left, anterior). (G) Graph showing the relative expression of h2b, as determined by qRT-PCR, in regenerating trunk fragments. Values represent the means of three biological replicates. (H) Fluorescence-associated cell sorting (FACS) analysis showing the proportion of distinct cell populations at different time points during regeneration. Values represent the means of at least two biological replicates. The pH3 and h2b images correspond to confocal z-projections. Error bars represent the standard error of the mean. Data were analyzed by Student's t-test. *P<0.05; **P<0.01; differences are considered significant at P<0.05. Scale bars: 300 µm. hR, hours of regen [file pgen.1004400.s005.tif]

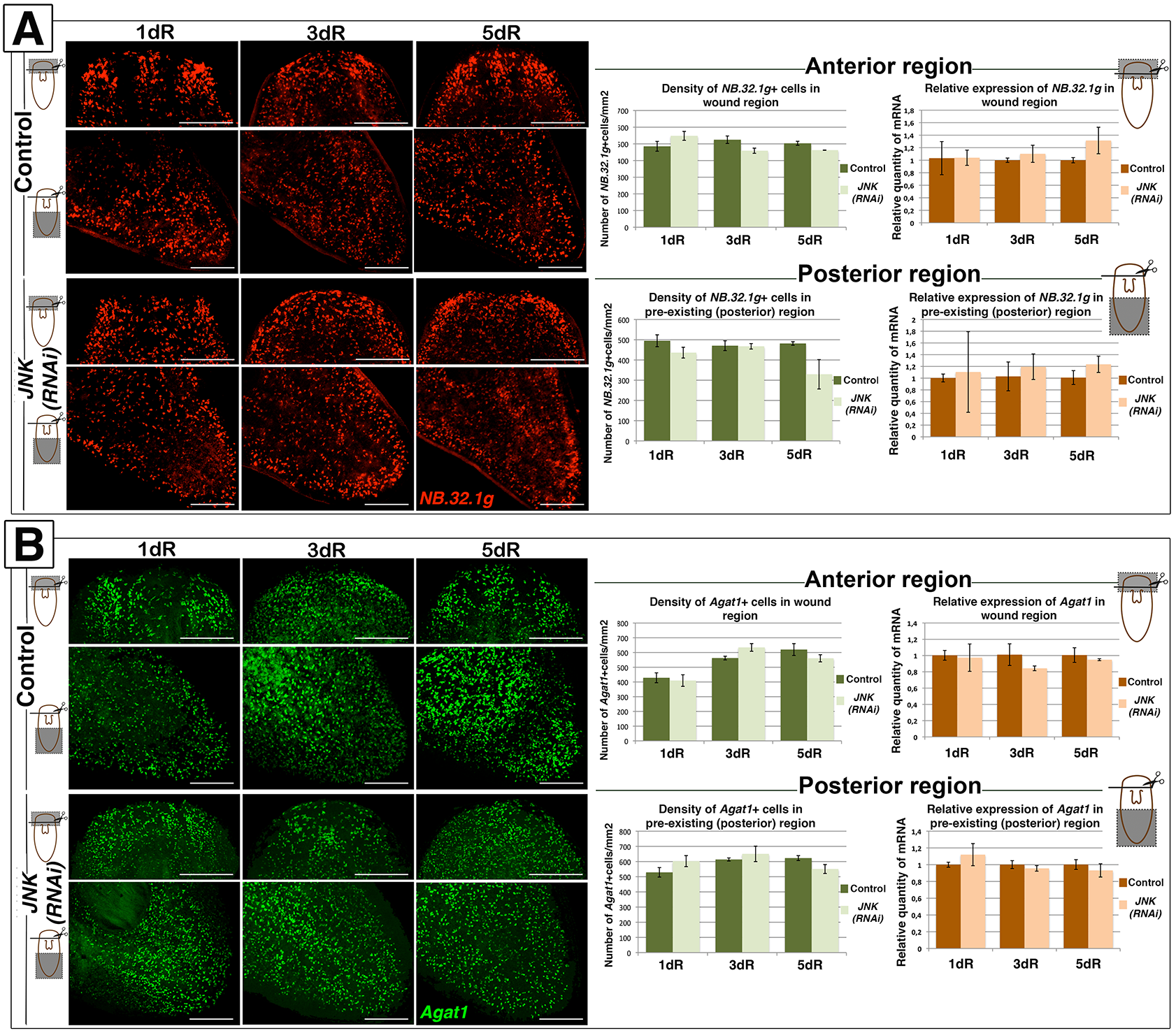

Supplement: Figure S6 — The numbers of early and late neoblast progeny cells are maintained after JNK RNAi in both regenerating and pre-existing regions. (A) FISH showing the expression of NB.32.1g, a marker of early post-mitotic descendants of neoblasts, in regenerating trunk fragments after anterior amputation. Images of the wound and the post-pharyngeal (pre-existing) region are shown. The green histogram depicts the quantification of NB.32.1g+ cells after FISH. At least four biological replicates were used per time point. The orange histogram depicts the relative expression of NB.32.1g as determined by qRT-PCR. Values represent the means of three biological replicates. Analysis from wound and post-pharyngeal (pre-existing) regions are shown. (Top/top left, anterior). (B) FISH showing the expression of Agat-1, a marker of late post-mitotic descendants of neoblasts, in regenerating trunk fragments after anterior amputation. Images of the wound and post-pharyngeal (pre-existing) region are shown. The green histogram depicts the quantification of Agat-1+ cells after FISH. At least five biological replicates were used per time point. The orange histogram depicts the relative expression of Agat-1 as determined by qRT-PCR. Values represent the means of three biological replicates. Analysis from wound and post-pharyngeal (pre-existing) regions are shown. (Top/top left, anterior). All images correspond to confocal z-projections. Error bars represent the standard error of the mean. Data were analyzed by Student's t-test. Differences are considered significant at P<0.05. Scale bars: 200 µm. dR, days of regeneration. (TIF) [file pgen.1004400.s006.tif]

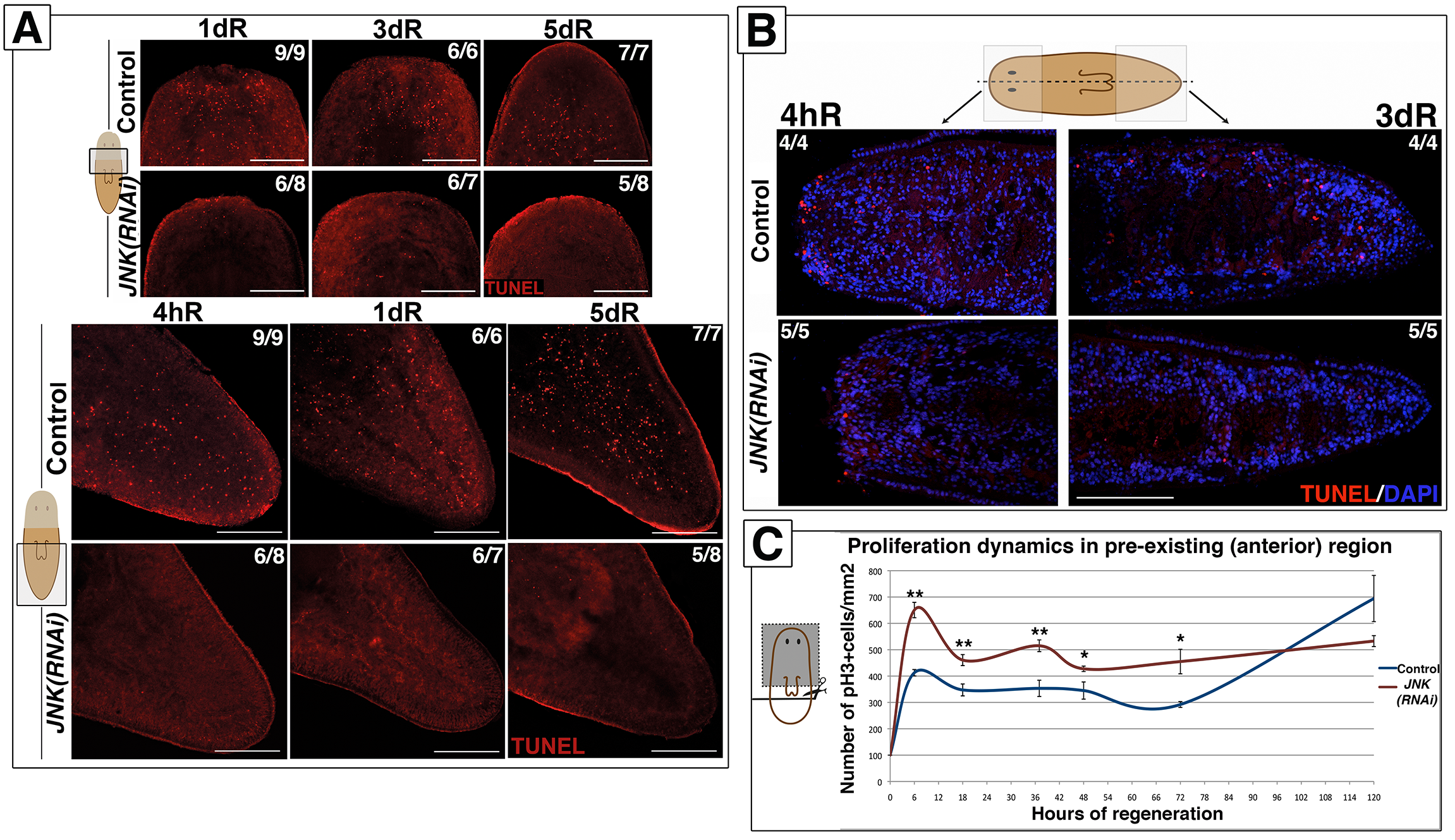

Supplement: Figure S7 — JNK plays a general pro-apoptotic role and coordinates the restoration of body proportion after any kind of amputation. (A) Whole-mount TUNEL staining showing apoptotic cell death in regenerating trunk fragments after anterior amputation. Images of the wound and post-pharyngeal (pre-existing) region are shown. (Top/top left, anterior). (B) TUNEL staining in longitudinal tissue sections showing apoptotic cell death in regenerating trunk fragments after anterior amputation. Images of the wound region 4 hours after amputation and of the post-pharyngeal (pre-existing) region 3 days after amputation are shown. (Left, anterior). (C) Graph showing the quantity of mitotic cells (pH3+) in anterior (pre-existing) regions of regenerating trunks after posterior amputation. At least four biological replicates were used per time point. All images correspond to confocal z-projections. Error bars represent the standard error of the mean. Data were analyzed by Student's t-test. *P<0.05; **P<0.01; Differences are considered significant at P<0.05. Scale bars: 200 µm. dR, days of regeneration. (TIF) [file pgen.1004400.s007.tif]

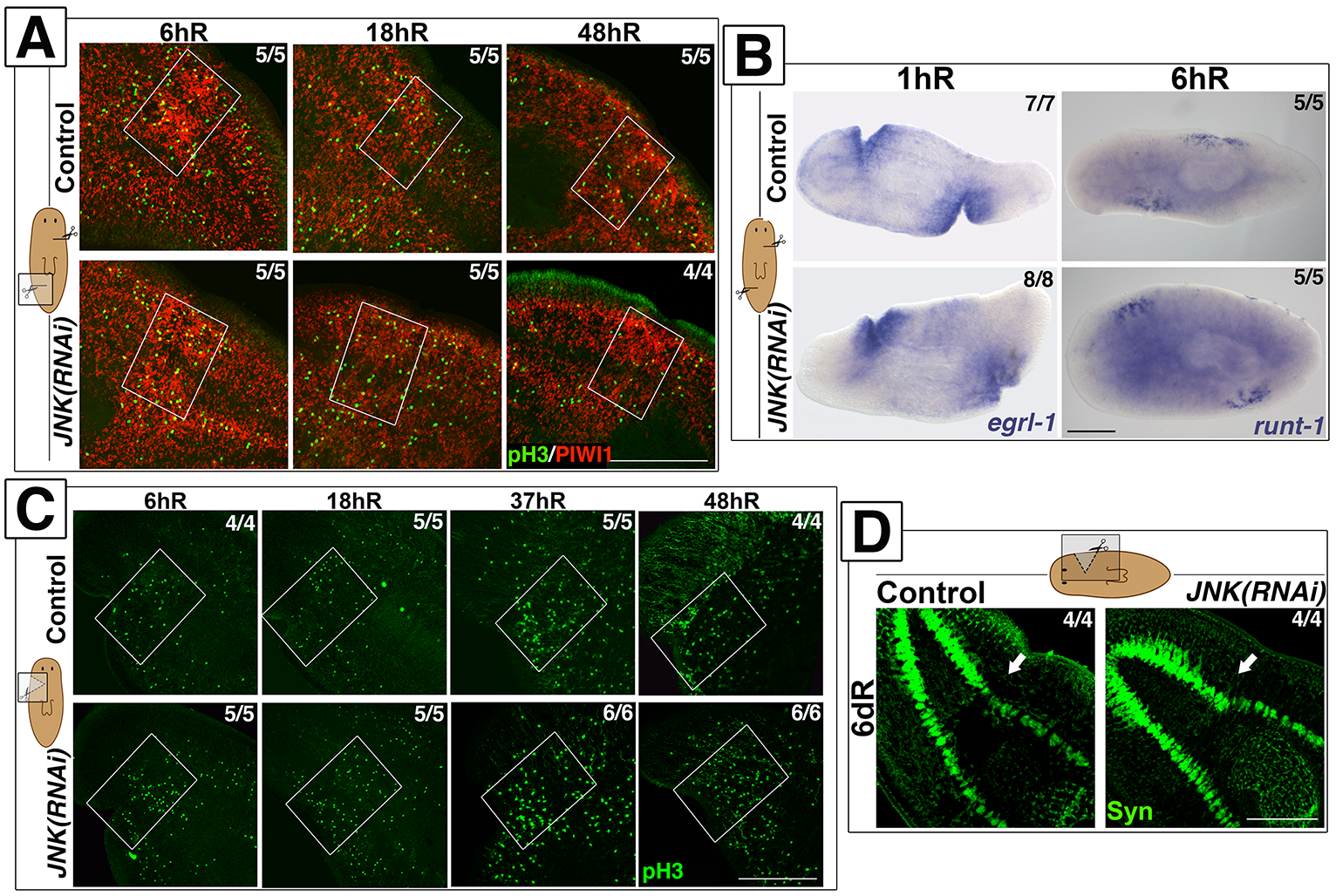

Supplement: Figure S8 — JNK is specifically required for de novo formation of new tissue after injury. (A) Anti-pH3 and anti-PIWI-1 immunostaining showing the dynamics of the mitotic response after a simple incision without loss of tissue. (Top left, anterior). (B) Analysis WISH of the genes expressed in response to incision. (Left, anterior). (C) Anti-pH3 immunostaining showing the dynamics of the mitotic response after a small lateral amputation. (Top left, anterior). (D) Anti-synapsin immunostaining demonstrating the regeneration of a previously amputated portion of ventral nerve cord (VNC). (Top left, anterior). The pH3/PIWI1, pH3 and Syn images correspond to confocal z-projections. Scale bars: 200 µm (A), 300 µm (B), 200 µm (C), 200 µm (D). hR, hours of regeneration; dR, days of regeneration. (TIF) [file pgen.1004400.s008.tif]

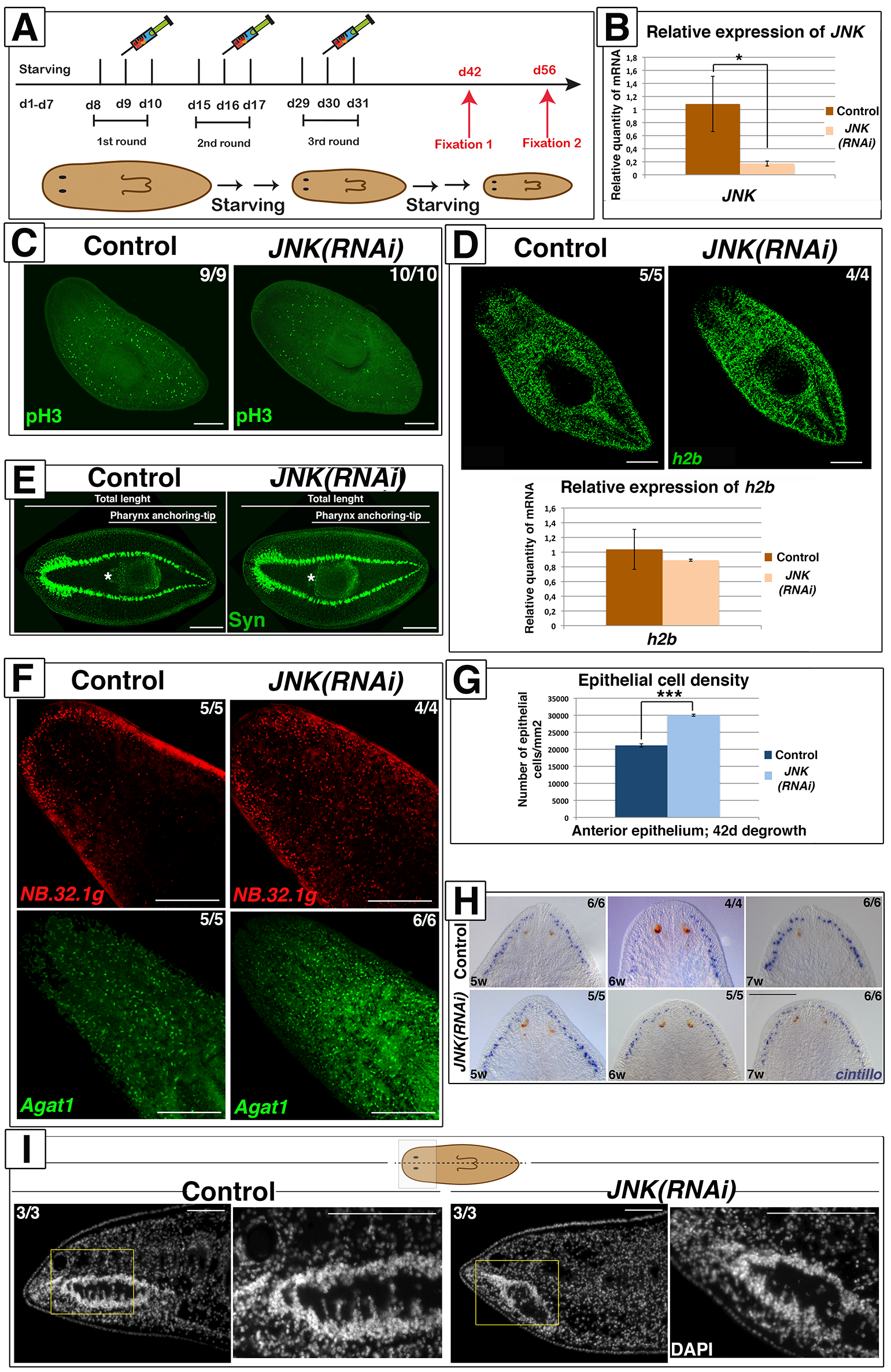

Supplement: Figure S9 — Role of JNK during homeostatic degrowth. (A) Cartoon illustrating the experimental design of the analysis of JNK function during degrowth. Animals were starved and injected with RNAi for three weeks to achieve reliable gene interference and were subsequently fixed after 42 or 56 days of starvation. (B) Graph showing the relative expression of JNK as determined by qRT-PCR in degrowing animals. JNK expression levels in JNK (RNAi) animals were significantly reduced, validating the gene interference approach. Values represent the means of three biological replicates. (C) Anti-pH3 immunostaining showing mitotic activity after 42 days of starvation. (Top left, anterior). (D) FISH showing the expression of h2b in animals starved for 42 days and graph showing the relative expression of h2b as determined by qRT-PCR in degrowing animals. Values represent the means of three biological replicates. (Top left, anterior). (E) Immunostaining with anti-synapsin to visualize the central nervous system and the pharynx of degrowing animals. The relative length of the posterior region was calculated by measuring the distance from the pharynx-anchoring to the tail tip and dividing this by the whole-body length. The white asterisk indicates the anchoring of the pharynx. (Left, anterior). (F) FISH showing the expression of NB.32.1g and Agat-1, markers of post-mitotic descendants of neoblasts, in degrowing animals. (Top left, anterior). (G) Graph illustrating the quantification of the number of epithelial cells/mm2 in the anterior regions of animals starved for 42 days. Eight biological replicates were used. (Top left, anterior). (H) WISH analysis of the anterior chemoreceptors (cintillo+) during starvation. (Top, anterior). (I) DAPI-stained tissue sections showing the brain of animals starved for 42 days. Corresponding images at increased magnification are shown aside. (Left, anterior). All images except for cintillo correspond to confocal z-projections. Error bars represent standard error [file pgen.1004400.s009.tif]

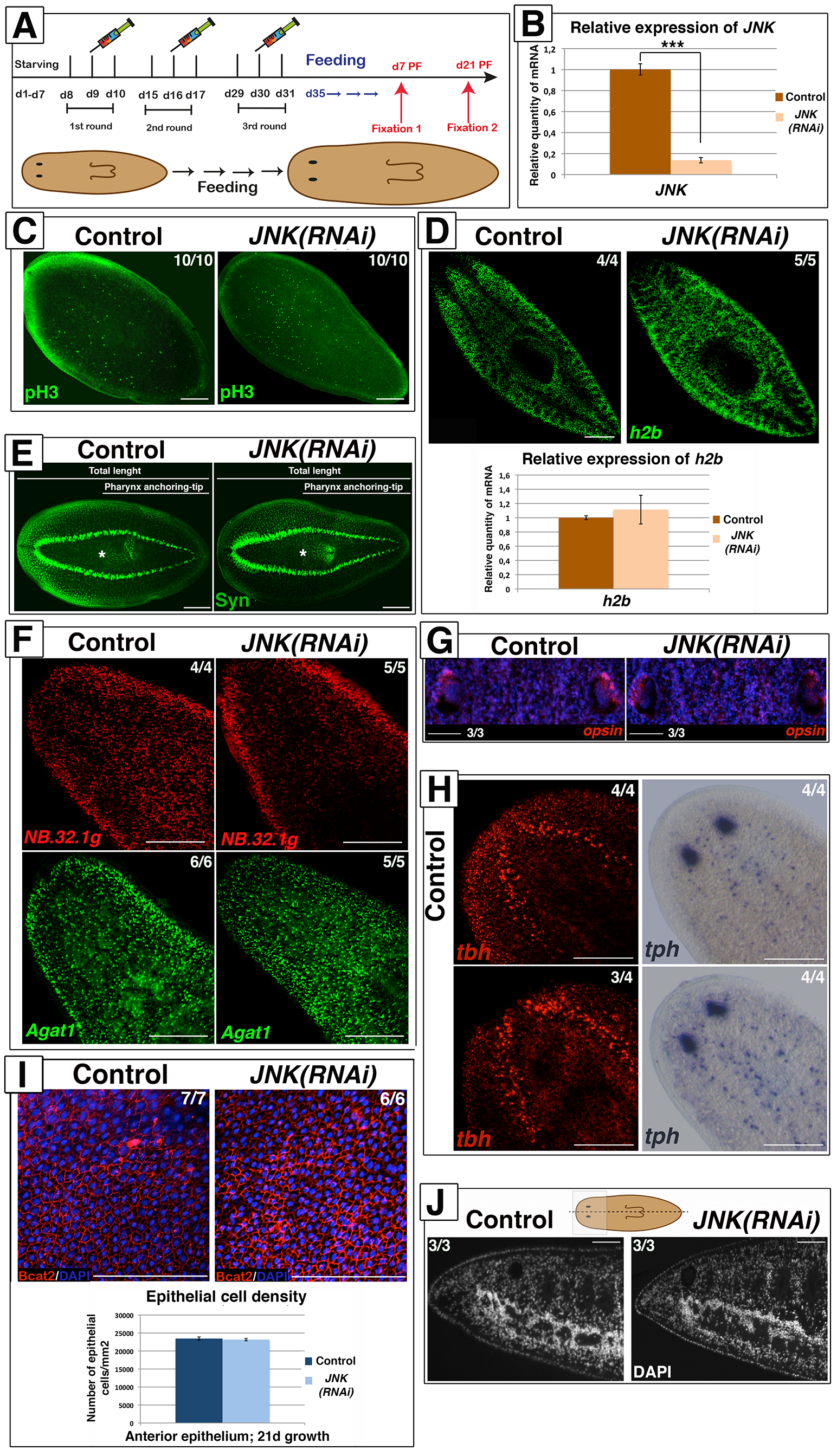

Supplement: Figure S10 — Role of JNK in homeostatic growth. (A) Cartoon illustrating the experimental design of the analysis of JNK function during growth. Animals were starved and injected with the RNAi for three weeks to achieve reliable gene interference. They were subsequently fed once every two days and were then fixed 7 or 21 days after the first feed. (B) Graph showing the relative expression of JNK in growing animals as determined by qRT-PCR. JNK expression levels in JNK(RNAi) animals were significantly reduced as compared with controls, validating the gene interference approach. Values represent the mean of three biological replicates. (C) Anti-pH3 immunostaining showing mitotic activity after 7 days of feeding. (D) FISH showing the expression of h2b in animals fed for 7 days and graph showing the relative expression of h2b as determined by qRT-PCR in growing animals. Values represent means of three biological replicates. (Top left, anterior). (E) Immunostaining with anti-synapsin to visualize the central nervous system and the pharynx of growing animals. The relative length of the posterior region was calculated by measuring the distance from the anchoring of the pharynx to the tail tip and dividing this by the whole-body length. White asterisk indicates the anchoring of the pharynx. (Left, anterior). (F) FISH showing the expression of NB.32.1g and Agat-1, markers of post-mitotic descendants of neoblasts, in growing animals. (G) FISH analysis of photoreceptor cells (opsin+) counterstained with DAPI (Top, anterior). (H) FISH analysis of octopaminergic (tbh+) neurons and WISH analysis of serotoninergic (tph+) neurons in the brains of animals fed for 21 days. (Top left, anterior). (I) Staining of the epithelia with DAPI and anti-β-catenin-2 antibody (Bcat2) and graph illustrating the quantification of the number of epithelial cells/mm2 in anterior regions from animals fed for 21 days. Six biological replicates were used. (Top left, anterior). (J) DAPI staining in tissue sections from [file pgen.1004400.s010.tif]
